# Baikalomycins A-C, New Aquayamycin-Type Angucyclines Isolated from Lake Baikal Derived *Streptomyces* sp. IB201691-2A

**DOI:** 10.3390/microorganisms8050680

**Published:** 2020-05-07

**Authors:** Irina Voitsekhovskaia, Constanze Paulus, Charlotte Dahlem, Yuriy Rebets, Suvd Nadmid, Josef Zapp, Denis Axenov-Gribanov, Christian Rückert, Maxim Timofeyev, Jörn Kalinowski, Alexandra K. Kiemer, Andriy Luzhetskyy

**Affiliations:** 1 Institute of Biology, Irkutsk State University, 664003 Irkutsk, Russia; irina.voytsekhovskaya@gmail.com (I.V.); denis.axengri@gmail.com (D.A.-G.); m.a.timofeyev@gmail.com (M.T.); 2 Helmholtz Institute for Pharmaceutical Research Saarland, 66123 Saarbrücken, Germany; Constanze.Paulus@helmholtz-hzi.de; 3 Pharmaceutical Biology, Saarland University, 66123 Saarbrücken, Germany; charlotte.dahlem@uni-saarland.de (C.D.); j.zapp@mx.uni-saarland.de (J.Z.); pharm.bio.kiemer@mx.uni-saarland.de (A.K.K.); 4 Pharmaceutical Biotechnology, Saarland University, 66123 Saarbrücken, Germany; y.rebets@mx.uni-saarland.de (Y.R.); suvdn@yahoo.com (S.N.); 5 Baikal Research Centre, 664003 Irkutsk, Russia; 6 Technology Platform Genomics, Center for Biotechnology (CeBiTec), Bielefeld University, 33615 Bielefeld, Germany; Christian.Rueckert@cebitec.uni-bielefeld.de (C.R.); joern@cebitec.uni-bielefeld.de (J.K.)

**Keywords:** natural products, angucycline, aquayamycin, glycosyltransferase, Lake Baikal, *Streptomyces*

## Abstract

Natural products produced by bacteria found in unusual and poorly studied ecosystems, such as Lake Baikal, represent a promising source of new valuable drug leads. Here we report the isolation of a new *Streptomyces* sp. strain IB201691-2A from the Lake Baikal endemic mollusk *Benedictia baicalensis*. In the course of an activity guided screening three new angucyclines, named baikalomycins A–C, were isolated and characterized, highlighting the potential of poorly investigated ecological niches. Besides that, the strain was found to accumulate large quantities of rabelomycin and 5-hydroxy-rabelomycin, known shunt products in angucyclines biosynthesis. Baikalomycins A–C demonstrated varying degrees of anticancer activity. Rabelomycin and 5-hydroxy-rabelomycin further demonstrated antiproliferative activities. The structure elucidation showed that baikalomycin A is a modified aquayamycin with β-d-amicetose and two additional hydroxyl groups at unusual positions (6a and 12a) of aglycone. Baikalomycins B and C have alternating second sugars attached, α-l-amicetose and α-l-aculose, respectively. The gene cluster for baikalomycins biosynthesis was identified by genome mining, cloned using a transformation-associated recombination technique and successfully expressed in *S. albus* J1074. It contains a typical set of genes responsible for an angucycline core assembly, all necessary genes for the deoxy sugars biosynthesis, and three genes coding for the glycosyltransferase enzymes. Heterologous expression and deletion experiments allowed to assign the function of glycosyltransferases involved in the decoration of baikalomycins aglycone.

## 1. Introduction

Angucyclines are by far the largest group of aromatic polyketides solely produced by actinobacteria [1,2]. These natural products have diverse biological activities, including antibacterial, anticancer, antiviral, enzyme inhibitory, fungicidal, and others. Angucyclines are assembled by the repetitive condensation of malonyl-CoA to produce a common benz[a]anthracene intermediate [2]. This reaction is performed by type II polyketide synthase enzymes. Two pathways to form the benz[a]anthracene aglycone of angucyclines exist. In most cases, the initial decaketide chain undergoes a series of cyclizations and aromatizations dictated by cyclases/aromatases to produce an angular core structure [3]. This major route takes place in the biosynthesis of vineomycins [4], jadomycins [5], and landomycins [6], for example. However, at least in two cases, BE-7585A and PD116198, the polyketide chain is folded into an anthracyclinone intermediate that further undergoes oxidative A-ring opening and rearrangement into an angular benz[a]anthracene structure [7,8]. The first common benz[a]anthracene intermediate for both routes, UWM6, can be preserved or further modified by a series of oxidation and reduction events and decorated by extensive glycosylation or other modifications [2]. Glycosylation is a distinctive feature of many angucyclines sharing a common aglycone structure. The length and composition of oligosaccharide chains have a strong impact on biological activities [9].

**Figure 1:**
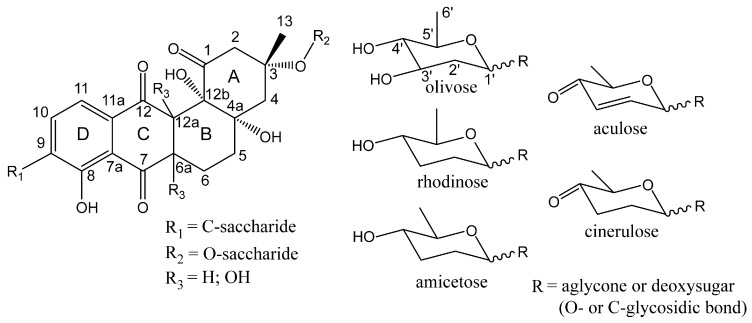
Structures of an aglycone and deoxy sugars typical for the aquayamycin-type angucyclines. Carbon atoms of the aglycone and sugars (with ′) are labeled according to IUPAC rules. The rings of the aglycone are indicated as A–D. The compound with R_1_ as a d-olivose is historically considered as a common “aquayamycin-type aglycone”.

Saquayamycins are a large group of angucyclines that, together with urdamycins, derive from the aquayamycin-type aglycone (Figure 1) [10]. More than ten derivatives of saquayamycins are known that mainly differ by the glycosylation pattern [4,11,12,13,14,15]. The largest representative of this group of natural products, saquayamycin Z, contains nine sugars [16]. Vineomycins and grincamycins as well as recently discovered Sch 47554–47555 and saprolmycins A–E also possess an aquayamycin-type aglycone [17,18]. Several other natural products have a modified aquayamycin-like aglycone structure, namely moromycins A and B and N05WA963 A–C, that lack the angular hydroxyl groups at positions 4a and 12b, resulting in a fully aromatic ring B [19,20]. N05WA963 A–C possesses an additional methoxy group at C-5. Lastly, several natural products with an opened ring A are known to derive directly from aquayamycin-type angucyclines. These include fridamycins A–E, himalomycins A and B, vineomycin B2, amicenomycins A, and the recently discovered vineomycin D [21,22,23]. It is still under discussion if these compounds are true naturally occurring products of biosynthetic pathways or are derived from the acid hydrolysis of respective angucyclines [21,24,25,26,27].

The distinct feature of the aquayamycin-type angucyclines is the presence of oligosaccharide chains attached at positions C-9 and C-3 (Figure 1). Typically, the first sugar at the C-9 position is C-linked d-olivose as in the case of saquayamycins, vineomycins, moromycins, grincamycins and N05WA963 A–C. At the same time, saquayamycins, vineomycins, moromycins, and grincamycins at C-3 have *O*-glycosidically attached l-rhodinose. N05WA963 A–C do not have a saccharide chain at C-3 [20]. Differences in the length and composition of the attached oligosaccharide chains determine the variety of aquayamycin-type angucyclines. Saquayamycins (except saquayamycin Z) and moromycins have diverse disaccharides at C-3 and C-9 [11,15,19]. Grincamycins and vineomycins have a trisaccharide at C-9 consisting of d-olivose, l-rhodinose and the ketosugar l-aculose or l-cinerulose (aculose derivative) [14,21]. A similar glycosylation pattern was observed in saprolmycins A–E with either trisaccharide or disaccharide at C-9 and l-aculose or l-cinerulose as a first and the only sugar at the C-3 position [18]. Sch 47554 and Sch 47555 also contain l-aculose at C-3 but carry C-bound d-amicetose (stereoisomer of rhodinose) at C-9 extended with either l-amicetose or l-aculose [17]. Lastly, amicenomycins A and B have the trisaccharide l-amicetose-l-amicetose-l-rhodinose at C-3 and a single d-olivose at C-9 [22]. As can be seen, despite the common aglycone, aquayamycin-type angucyclines represent a diverse group of natural products due to differences in the glycosylation pattern.

The glycosylation events are well studied in the case of saquayamycin Z and saquayamycins G-K [16,28]. The saquayamycins G–K biosynthetic gene cluster (*sqn*) contains three genes *sqnGT1*–*3* encoding glycosyltransferases. However, only SqnGT2 was shown to be essential for decorating the aglycone, while the other two, SqnGT1 and SqnGT3, are proposed to act as chaperons, modulating the SqnGT2 activity [28]. Similarly, the Sch 47554 and Sch 47555 biosynthetic gene cluster also encode three glycosyltransferases (*schS7*, *schS9* and *schS10*) [29]. Genetic studies have shown that SchS7 attaches d-amicetose at C-9 and SchS9 further extends the saccharide chain when SchS10 attaches l-aculose at C-3 position [30]. Gene clusters for saprolmycins (*spr*) and grincamycins (*gcn*) biosynthesis have been recently cloned and were found to contain three genes encoding glycosyltransferases [27,31]. This implies that the different decoration pattern of these angucyclines results from the differences in functional properties of the glycosylation enzymes.

Here we report the characterization of the new aquayamycin-type angucycline antibiotics baikalomycins A–C produced by *Streptomyces* sp. IB201691-2A. The strain was isolated from the Lake Baikal endemic gastropod *Benedictia baicalensis*. Baikalomycins demonstrated moderate anticancer and antibacterial activities. The genome sequencing and mining led to identification of a gene cluster responsible for the biosynthesis of baikalomycins. Heterologous expression and gene deletion experiments supported this finding and provided hints on the glycosylation steps in baikalomycins biosynthesis.

## 2. Materials and Methods

### 2.1. Bacterial Strains, Culture Conditions and Routine Procedures

*Streptomyces* sp. IB201691-2A and *Rhodococcus* sp. IB201691-2A2 were isolated during this work. *Streptomyces albus* J1074 was used as a host for the heterologous expression of the baikalomycin biosynthetic gene cluster [32]. For the routine cloning, *Escherichia coli* XL1Blue (Agilent, Santa Clara, CA, USA) has been used and intergenic conjugation was carried out with *E. coli* ET12567 (pUB307) [33]. *S. cerevisiae* BY4742 was used for transformation-associated recombination cloning [34]. *E. coli* strains were grown in Luria–Bertani (LB) broth. Actinobacteria strains were cultured on soya flour mannitol agar (MS) medium and in liquid tryptic soy broth medium (TSB; Sigma-Aldrich, St. Louis, MO, USA). If necessary, the following antibiotics have been added: apramycin (50 µg·mL^−1^), spectinomycin (100 µg·mL^−1^), phosphomycin (100 µg·mL^−1^) and carbenicillin (100 µg·mL^−1^) (Sigma-Aldrich, St. Louis, MO, USA; Roth, Karlsruhe, Germany). The chromogenic substrate X-gluc with 100 µg·mL^−1^ concentration was used to detect the GUS (β-glucuronidase) activity.

Plasmid and total DNA isolation, *E. coli* transformation and *E. coli*/*Streptomyces* intergeneric conjugation were performed according to standard protocols [35,36]. *S. cerevisiae* BY4742 was transformed with the standard LiAc protocol [36]. Enzymes, including restriction endonucleases, ligase, Taq DNA polymerase, Klenow fragment of DNA polymerase I, were used according to manufacturer’s recommendations (New England Biolabs, Ipswich, MA, USA; Thermo Fischer Scientific, Waltham, MA, USA; Agilent, Santa Clara, CA, USA).

### 2.2. Sampling and Actinobacteria Isolation

Endemic mollusks *Benedictia baicalensis* (Gerstfeldt, 1859) [37] were collected from Lake Baikal near Bolshiye Koty village (51°54′19″ N 105°4′31″ E, western shore of Lake Baikal) at depths of 50 and 100 m in February 2016 using deep-water traps. Each mollusks’ sample included up to 5 specimens. Mollusks were surface-washed with sterile water, 70% ethanol, and again with sterile water to eliminate transient microorganisms. Afterwards, prepared samples were homogenized in 20% sterile glycerol and stored at −20 °C. Homogenates were thawed on ice and plated on MS plates supplemented with phosphomycin (50 µg/mL) and cycloheximide (100 µg/mL). Plates were incubated at 28 °C for 14 days. Colonies with typical for actinobacteria morphology were picked on a fresh MS plate and further characterized.

### 2.3. 16S rRNA Gene Sequencing and Phylogenetic Analysis

Strains were grown in 10 mL of TSB medium at 28 °C for 3 days and 180 rpm and total DNA was isolated using standard method [35]. The *16S* rRNA gene was amplified by PCR with the modified universal 8F and 1492R primers (Supplementary Table S1) [38]. PCR was carried out with initial denaturation at 95 °C for 3 min, followed by 30 cycles of 95 °C for 35 s, 51 °C for 40 s and 72 °C for 110 s, with an end extension at 72 °C for 7 min. The PCR products were purified using the Wizard SV Gel and PCR Clean-Up System (Promega, Madison, WI, USA) and sequenced using 8F and 1492R primers (Supplementary Table S1) [38]. The forward and reverse sequences were assembled with Bioedit software (version 7.2.5, Tom A. Hall, Department of Microbiology, North Carolina State University, North Carolina, USA, freeware). Evolutionary analyses were conducted in MEGA7 using *16S* rRNA gene sequences of related strains (Supplementary Table S2) [39]. The evolutionary history was inferred using the neighbor-joining method [40].

### 2.4. Screening the Culture Conditions for Biological Activity of Streptomyces sp. IB2016I91-2A

The following media were used for metabolites production by *Streptomyces* sp. IB2016I91-2A: SM1 (soy flour 10 g, glucose 18 g, Na_2_SO_4_ 1 g, CaCO_3_ 0.2 g, pH 7.0, 1 L tap water), SM17 (soy flour 5 g, glucose 2 g, glycerol 40 g, soluble starch 2 g, peptone 5 g, yeast extract 5 g, NaCl 5 g, CaCO_3_ 2 g, pH 6.4, 1 L tap water), SM12 (soy flour 10 g, glucose 50 g, peptone 4 g, meat extract 4 g, yeast extract 1 g, NaCl 2.5 g, CaCO_3_ 5 g, pH 7.6, 1 L tap water), SM20 (maltose 20 g, peptone 5 g, meat extract 5 g, yeast 3 g, MgSO_4_ × 7 H_2_O 1 g, NaCl 3 g, pH 7.2, 1 L tap water), SM24 (yeast extract 9 g, peptone 1.8 g, glucose 20 g, KH_2_PO_4_ 1 g, MgSO_4_ × 7 H_2_O 0.5 g, pH 6.2, 1 L distilled water), SM25 (peptone 10 g, malt extract 21 g, glycerol 40 g, pH 6.5, 1 L distilled water), SM27Ac (soy flour 10 g, glucose 50 g, peptone 4 g, meat extract 4 g, yeast extract 1 g, NaCl 2.5 g, CaCO_3_ 5 g, soluble starch 5 g, pH 4.5 with HCl, 1 L tap water), SM27N and SM27A1 (SM27Ac at pH 7.0 and pH 8.7 with NaOH respectively), R2 (malt extract 10 g, yeast extract 4 g, glucose 4 g, artificial sea water 0.5 L, pH 7.8, 0.5 L tap water), and Hopwood minimal medium [35]. The strain was inoculated into 50 mL of TSB medium in 500 mL flasks and grown for 3 days at 28 °C on a rotary shaker at 180 rpm. An inoculation was carried out of 5 mL of seed culture into 50 mL of production media and incubated for 8 days at 28 °C on a rotary shaker at 180 rpm. The metabolites from cultural broth were extracted with equal volume of ethyl acetate. Organic solvent was evaporated and the obtained extracts were dissolved in 500 µL of methanol.

### 2.5. LC-MS and LC-HRMS Analysis

LC-MS (Liquid chromatography–mass spectrometry) measurements were performed on a Dionex Ultimate 3000 RSLC (Thermo Fischer Scientific, Waltham, MA, USA) system using a BEH C18, 100 × 2.1 mm, 1.7 µm *d*_p_ column (Waters, Eschborn, Germany). Injection volume amounts to 1 µL and elution was achieved by a linear gradient (5–95% over 18 min) of solvent B (distilled acetonitrile with 0.1% of formic acid) against solvent A (bi-distilled water with 0.1% of formic acid). The column thermostat was set to 45 °C and a flow rate of 600 µL/min was used. UV spectra were recorded using DAD detector in the range of 200–600 nm and mass spectrometry data were collected on amazon SL speed (Bruker, Billerica, MA, USA) with an Apollo II ESI source in a range of 200–2000 *m*/*z*. High-resolution mass spectroscopic data (HRMS) with LC were acquired on a Dionex Ultimate 3000 RSLC system (Thermo Fischer Scientific, Waltham, MA, USA) using a BEH C18, 100 × 2.1 mm, 1.7 µm *d*_p_ column (Waters, Eschborn, Germany). A linear gradient from 5–95% solvent B (distilled acetonitrile + 0.1% formic acid) against solvent A (bi-distilled water + 0.1% formic acid) at a flow rate of 450 µL/min and 45 °C column temperature was used to separate 1 µL sample. UV spectroscopic data were collected by a DAD detector in the range of 200–600 nm. Mass spectroscopic data were acquired with an LTQ Orbitrap mass spectrometer (Thermo Fischer Scientific, Waltham, MA, USA). LC-MS data were collected, processed, and analyzed with Bruker Compass Data Analysis software, version 4.2 (Bruker, Billerica, MA, USA) and the Thermo Xcalibur software, version 3.0 (Thermo Fischer Scientific, Waltham, MA, USA). Dereplication was carried out by means of the Dictionary of Natural Products Database, version 10.0 (CRC Press, Baca Raton, FL, USA) with accurate mass, UV absorption maxima, and biological source as parameters [41].

### 2.6. Isolation and Purification of Compounds 1–5

For purification of the angucyclines, the strain *Streptomyces* sp. IB2016I91-2A was cultivated in 10 L of SM27N medium (pH 7.0), as described above. The metabolites were extracted with ethyl acetate from the cultural liquid and solvent was removed under reduced pressure. The obtained extract, 2.04 g, was dissolved in 13 mL of methanol and subjected to size-exclusion chromatography. The crude extract was loaded on a glass column (1 m) packed with Sephadex^®^ LH 20 (total volume ~ 700 mL; Sigma-Aldrich, St. Louis, MO, USA). Methanol was used as eluent and fractions were collected every 15 min with a speed of 1–2 drops per second. Fractions were analyzed on LC-MS and targeted fractions further purified through preparative and semipreparative high performance liquid chromatography (HPLC) with the following equipment. A preparative HPLC system the Dionex Ultimate 3000 (Thermo Fischer Scientific, Waltham, MA, USA) equipped with a Nucleodur C18 HTEC column (150 × 21 mm, 5 µm) and linear gradient from 5–95% solvent B (acetonitrile + 0.1 formic acid) against solvent A (water + 0.1 % formic acid) over 28 min with a flow rate of 17 mL/min was used for initial purification. Obtained fractions containing the targeted compounds were further purified on a semipreparative HPLC system Agilent 1260 Series (Agilent Technologies, Santa Clara, CA, USA). Compounds 1, 2, and 5 were purified using Jupiter proteo C12 column (250 × 10 mm, 4 µm; Phenomenex, Madrid Ave, Torrance, CA, USA). Compounds 1 and 2 were obtained using a gradient starting from 30% of solvent B (acetonitrile + 0.1 formic acid, A: water + 0.1 % formic acid) and an increase of solvent B to 95% over 25 min. For compound 5, a multistep gradient from 5–75% B over 8 min and an increase to 85% B over 24 min was used. Compounds 3 and 4 were separated on the same system with Synergy Fusion RP column (250 × 10 mm, 4 µm; Phenomenex, Madrid Ave, Torrance, CA, USA) using a multistep gradient starting from 5% B (acetonitrile + 0.1% formic acid, A: water + 0.1% formic acid) to 45% over 17 min and a further increase to 95% B over 8 min. In a second step, compounds 3 and 4 were purified once more using the same column and a multistep gradient from 5–50% B over 10 min and an increase to 70% over 15 min. In case of compounds 1, 2, and 5, a flow rate of 5.0 mL/min and for compounds 3 and 4, a flow rate of 4.0 mL/min were used. Column thermostat was set to 45 °C and UV spectra were recorded in the range of 200–600 nm with DAD (Diode-Array Detector) detector.

NMR (Nuclear magnetic resonance) spectra were recorded in deuterated methanol (CD_3_OD) and deuterated dimethyl sulfoxide (DMSO-*d*_6_) at 298 K on a Bruker Avance III spectrometers (700 and 500 MHz; Bruker, MA, USA), both equipped with a 5 mm TXI cryoprobe. NMR data were analyzed using Topspin, version 3.5 pl7 (Bruker, Billerica, MA, USA).

### 2.7. Genome Sequencing and Bioinformatics

For isolation of total DNA, *Streptomyces* sp. IB2016I91-2A was grown in R5A medium [42] at 28 °C on a rotary shaker (180 rpm) for four days and a salting out procedure was used to obtain DNA [35]. For genome sequencing, an Illumina paired-end sequencing library (TruSeq sample preparation kit; Illumina, USA) was constructed as recommended by the manufacturer. The draft genome sequence was achieved on an Illumina MySeq system in rapid run mode (2 × 250 nt) with a pair distance of 500 bp. Subsequent to sequencing, the processed data were subjected to *de novo* assembly using SPAdes (version 3.8.1) [43] with default settings. Genome annotation was carried out using prokka v1.11 and GenDB 2.0 platform [44,45]. Secondary metabolism gene clusters were analyzed by the genome mining tool antiSMASH [46]. The genome sequence of *Streptomyces* sp. IB2016I91-2A was deposited under accession number SPQF00000000 in GenBank database.

### 2.8. Gene Disruption of the Glycosyltransferase Genes baiGT2 and baiGT3 

For deletion of *baiGT2*, the fragment 3L (2.485 kb) and the fragment 4R (2.366 kb) were amplified by PCR with primer pairs 3L-FHindIII and 3L-REcoRV, 4R-FEcoRV and 4R-RXbaI, respectively (Supplementary Table S1). For deletion of *baiGT3*, the fragment 5L (2.366 kb) and 6R (2.574 kb) were amplified with primer pairs 5L-FHindIII and 5L-REcoRV, 6R-FEcoRV and 6R-RXbaI, respectively. Obtained PCR fragments were cloned into a pJET1.2/blunt cloning vector (Thermo Fischer Scientific, Waltham, MA, USA) resulting in the plasmids pJET3L and pJET4R, and pJET5L and pJET6R. The 4R fragment was retrieved with *Eco*RV and *Xho*I and ligated into pJET3L digested with the same enzymes, giving pJET34. The 5L fragment was retrieved with *Eco*RV and *Xba*I and ligated into *Eco*RV-*Xba*I digested pJET6R, resulting in pJET56. The spectinomycin resistance gene *aadA* was obtained from pHP45Ω as *Eco*RV fragment and cloned into *Eco*RV digested pJET34 to yield pJET34sp and pJET56 to yield pJET56sp. The resulting plasmids were digested with *Hind*III, fragments that corresponded to deletion constructs 34 sp and 56 sp were gel-purified and treated with Klenow fragment of *E. coli* DNA polymerase I (New England Biolabs, Ipswich, MA, USA) and sub-cloned into pKG1132 vector digested with *Eco*RV creating the final constructs pKG1132sp-34 and pKG1132sp-56. The plasmids were introduced into *Streptomyces* sp. IB201691-2A using intergeneric conjugation. Exconjugants were screened for white spectinomycin-resistant colonies on MS plates supplemented with X-gluc and spectinomycin. The deletion of *baiGT2* and *baiGT3* genes was confirmed by PCR using the CheckGT2F and CheckGT2R, and the CheckGT3F and CheckGT3R primer pairs (Supplementary Table S1)

### 2.9. Cloning of the bai Gene Cluster Using Transformation-Associated Recombination (TAR) Technique

The two 2.5 kb (TAR1) and 2.4 kb (TAR2) DNA fragments flanking the 44 kb *bai* biosynthetic gene cluster were amplified using primer pairs 91-2aTAR1-FNotI /91-2aTAR1-RNheI and 91-2aTAR2-FNheI/91-2aTAR2-RHindIII (Supplementary Table S1), respectively, and cloned into pJET1.2/blunt cloning vector (Thermo Fischer Scientific, USA). The TAR1 was retrieved with *Hind*III and *Nhe*I and ligated into *Nhe*I/*Hind*III digested pJetTAR2. The resulting construct was digested by *Hind*III/*Not*I and sub-cloned into a pCLY10 vector [47]. The final construct was linearized with *Nhe*I and co-transformed into *S. cerevisiae* BY4742 with *Streptomyces* sp. IB201691-2A chromosomal DNA in ratios 1:1, 1:2, 1:3, 1:5. Transformants were grown onto the selection medium YNB supplemented with yeast synthetic drop-out medium supplements without leucine containing 1% of glucose for 4 days (Sigma-Aldrich, St. Louis, MO, USA). Yeast colonies were screened by PCR using the primers 91-2aCheck-FNotI and 91-2aCheck-RHindIII and pCLY10-RNotV (Supplementary Table S1). Total DNA was isolated from the positive clone pCLY8.13-10bai and transformed into *E. coli* XL1Blue. The plasmid containing the cloned *bai* cluster was named p8-13bai and verified by digesting with restriction enzymes *Eco*RI, *Xho*I, *Not*I and *Kpn*I. p8-13bai was introduced into *S. albus* J1074 via intergeneric conjugation. The recombinant *S. albus* J1074/p8-13bai was cultivated in SM27N and extracts were analyzed for production of baikalomycins as described above.

### 2.10. Biological Activity Assays

The biological activity of the crude extracts from small-scale cultivation of *Streptomyces* sp. IB2016I91-2A was screened using a disk diffusion assay. The 6 mm paper disks were loaded with 40 µL of each extract and dried. *Bacillus subtilis* ATCC 6633, *Pseudomonas putida* KT 2440, *Escherichia coli* K 12 were grown in liquid LB medium and *Saccharomyces cerevisiae* BY4742 in YPD. Test cultures were spread on solid LB and YPD medium and dried paper discs were placed on top. The plates were incubated at 37 °C for 12 h and 30 °C for 2 days (in case of *Saccharomyces cerevisiae*) and zones of inhibition were measured manually.

The minimal inhibitory concentration (MIC) was determined against the Gram-positive bacteria *Staphylococcus carnosus* DSM 20501 and *Mycobacterium smegmatis* DSM 43286, against the Gram-negative bacteria *Erwinia persicina* DSM 19328 and *Pseudomonas putida* KT2440, and against the yeast *Candida glabrata* DSM 11226. The minimal inhibitory concentrations were estimated by a standard serial dilutions protocol in 200 μL in 96-well plates using DMSO as solvent. Kanamycin was used as a positive control, and DMSO was used as negative control. 190 μL of bacterial test cultures in appropriate media (1:500 dilution of overnight culture) were added to each well containing 10 μL of compound solution. Plates were shaken at 30 °C for 16–20 h. To each well, 5 μL of thiazolyl blue tetrazolium bromide (10 mg/mL; Sigma-Aldrich, St. Louis, MO, USA) solution was added, and the plates were incubated at 30 °C for an additional 10–30 min. MICs were determined as the concentration of antibiotic in the well where the color of thiazolyl blue tetrazolium bromide was not changed from yellow to dark blue.

### 2.11. Anticancer Activities of Isolated Compounds

Assays were performed using the human tumor cell lines A549 (lung carcinoma), Huh7.5 (hepatocellular carcinoma), MCF7 (breast adenocarcinoma), and SW620 (colorectal adenocarcinoma). All cell lines were cultured in RPMI-1640 (A549, HuH7.5) or DMEM (MCF-7, SW620) supplemented with 10% fetal bovine serum, 100 U/mL penicillin, 100 mg/mL streptomycin, and 2 mM glutamine. The cells were maintained at 37 °C in a humidified atmosphere of 5% CO_2_.

A cell viability assay (MTT assay) was performed as described below. Cells were seeded in appropriate numbers to reach confluency the next day and were then treated with the respective compounds in different concentrations for 48 h. Stock solutions of the compounds were prepared in DMSO, and solvent controls were tested concurrently. The viability of adherent cells was determined by replacing the supernatants with 0.5 mg/mL MTT (3-(4,5-dimethylthiazole-2-yl)-2,5 diphenyltetrazolium bromide; Sigma-Aldrich, St. Louis, MO, USA) solution in respective culture media. After a 30 min incubation, the formazan crystals were dissolved in DMSO, and the absorbance was measured at 560 nm in a microplate reader (GloMax Discover, Promega, Madison, WI, USA). IC_50_ values were calculated by non-linear regression using OriginPro (OriginLab Corporation, Northampton, MA, USA).

A proliferation assay (ECIS) was performed as follow. Cell proliferation was measured using the electric cell-substrate impedance sensing (ECIS^®^) system (Applied BioPhysics, Road in Troy, NY, USA). On the day before cell seeding, the arrays were pre-incubated with full cell culture medium at 37 °C. A549 cells were grown on 96-well ECIS arrays (96W10E+, with 10 electrodes per well), and the impedance measurement (every 15 min for 100 h, 16,000 Hz) was started directly after cell seeding (8000 cells per well). The cells were left to attach for 5 h before the compounds were added to the cells at the indicated concentrations. Control cells were treated with the diluted solvent DMSO. Impedance was normalized to the value at 7 h after inoculation.

## 3. Results and Discussion

### 3.1. Isolation and Characterization of Streptomyces sp. IB201691-2A

**Figure 2:**
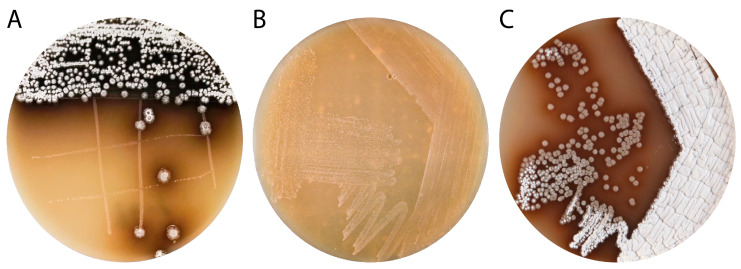
Actinobacteria species co-isolated from *Benedictia baicalensis* grown on soya flour mannitol (MS) agar. (**A**) Original mixture of *Streptomyces* sp. IB201691-2A and *Rhodococcus* sp. IB201691-2A2 obtained from a single colony grown from a plated homogenate of *B. baicalensis*; (**B**) Pure culture of *Rhodococcus* sp. IB201691-2A2; (**C**) Pure culture of *Streptomyces* sp. IB201691-2A.

The Lake Baikal endemic mollusk *Benedictia baicalensis* [37] was sampled (12× samples with five specimens, each) at a depth of 50 and 100 m in February 2016 at Lake Baikal in Bolshiye Koty village (51°54′19″ N 105°4′31″ E, western shore of Lake Baikal) and actinobacteria were isolated as described in the materials and methods section. Several actinobacteria-like colonies with similar morphology were found in three samples from 50 m and two samples from 100 m depth. After re-plating on fresh MS medium, all five isolates were noticed to contain two different species (Figure 2A). One of them demonstrated surface growth with pale orange colonies and did not form aerial mycelium and spores (Figure 2B). Based on *16S* rRNA gene sequence analysis, this bacterium was identified as a *Rhodococcus* sp. designated as IB201691-2A2 (Supplementary Figure S1).

The second bacterium has substrate growth typical for streptomycetes, forms white spores, and produces dark-brown pigment (Figure 2C). The 16S rRNA gene phylogeny analysis placed the strain into the genus *Streptomyces* and it was named *Streptomyces* sp. IB201691-2A (Supplementary Figure S2). Gene sequences of *16S* rRNA of *Rhodococcus* sp. IB201691-2A2 and *Streptomyces* sp. IB201691-2A specimens isolated from different samples were identical (data not shown). *Streptomyces* sp. IB201691-2A is closely related to *S. ederensis* NBRC 15410, producing moenomycins and *S. umbrinus* NBRC 13091 producing phaeochromycin and diumycins (moenomycin derivatives). Both strains were isolated from soil and are heterotypic synonyms of phaeochromycin-producing *S. phaeochromogenes* (Supplementary Figure S2) [48,49]. The *S. phaeochromogenes* strain was originally discovered as a producer of angucycline PD116198 [7]. In this study, we focused on *Streptomyces* sp. IB201691-2A strain.

### 3.2. Production and Isolation of Baikalomycins

For biological activity screening *Streptomyces* sp. IB201691-2A was cultured in nine different liquid media at three temperatures (13, 28, and 37 °C) and extracts were tested against four test-cultures, including Gram-positive and Gram-negative bacteria and yeast (Supplementary Table S3). The strain was found to produce compounds active only against *B. subtilis*. SM27N medium and 28 °C conditions were preferable for accumulation of these bioactive metabolites, as seen from the largest inhibition zone (Supplementary Table S3).

**Figure 3:**
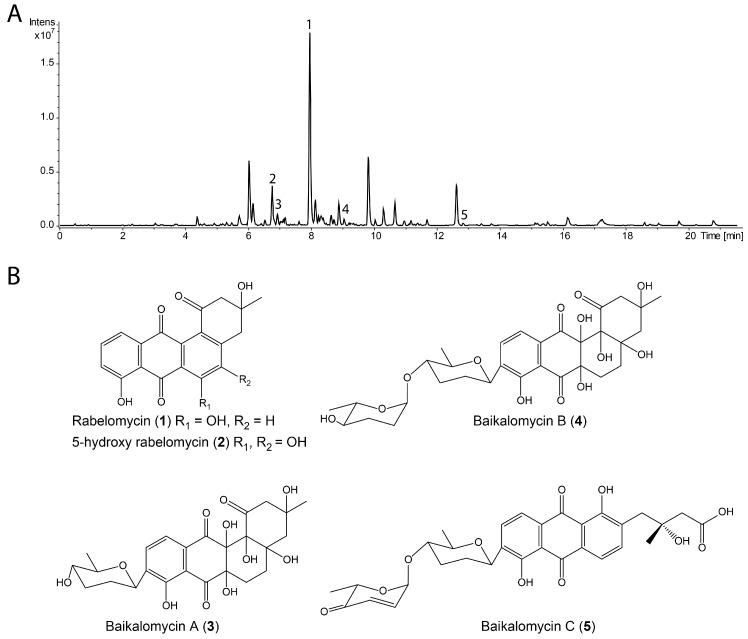
(**A**) LC chromatogram of crude extract of *Streptomyces* sp. IB201691-2A; (**B**) structure of compounds isolated from the extract of *Streptomyces* sp. IB201691-2A: rabelomycin (**1**) and 5-hydroxy-rabelomycin (**2**) and baikalomycins A-C (**3**–**5**).

The crude extract of *Streptomyces* sp. IB201691-2A grown in SM27N was analyzed by high-resolution LC-MS and dereplicated using the Dictionary of Natural Products database. Two known compounds rabelomycin (**1**) and 5-hydroxy-rabelomycin (**2**) were identified, with the former being the major product of the strain (Figure 3A,B; Supplementary Figures S3 and S4). Rabelomycin, a well-known shunt product in the biosynthesis of angucyclines, was also co-isolated together with vineomycins from extracts of *S. matensis* subsp. *vineus* and himalomycins A and B from *Streptomyces* sp. B6921 [4,25,50]. Besides these two compounds, several peaks with characteristics for angucyclines absorption spectra and *m*/*z* ranging from 330 to 620 (in negative mode) are present in the extract of *Streptomyces* sp. IB201691-2A. However, we were not able to identify these compounds within the Dictionary of Natural Products.

*Streptomyces* sp. IB201691-2A was cultivated in 10 L of the SM27N medium and metabolites were extracted, giving 2.04 g of a crude extract. Metabolites were fractionated by size exclusion chromatography and the targeted compounds were purified by the preparative HPLC. Five pure compounds were obtained (with 80–85% purity): Rabelomycin (**1**) (6.5 mg), 5-Hydroxy-rabelomycin (**2**) (1.8 mg), baikalomycin A (**3**) (RT 6.9 min, 0.9 mg), baikalomycin B (**4**) (RT 9.1 min, 1.1 mg) and baikalomycin C (**5**) (RT 12.8 min, 0.7 mg) (Figure 3A,B; Supplementary Figures S3–S5).

### 3.3. Structure Elucidation of Baikalomycins

The structures of rabelomycin (**1**) and 5-hydroxy-rabelomycin (**2**) were confirmed by the comparison of the ^1^H and ^13^C NMR data to the previously reported data (Figure 3B; Supplementary Table S4) [51,52].

**Figure 4:**
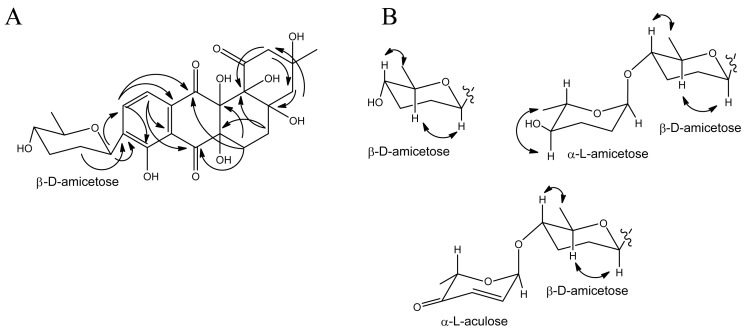
(**A**) Selected HMBC correlations within baikalomycin A; (**B**) Selected ROESY correlations which support the designated configuration of attached sugars.

Baikalomycin A (**3**) was obtained as a pale-yellow solid (purity > 80 mol%, according to ^1^H NMR, Supplementary Figure S6). The molecular formula of C_25_H_30_O_11_ and *m*/*z* 487.1634 [*M*-H_2_O-H]^−^ (calc. *m*/*z* 487.1604 [*M*-H_2_O-H]^−^) was determined on the basis of HRESIMS ( High-resolution electrospray ionisation mass spectrometry) data, indicating 11 degrees of unsaturation (Figure 3B; Supplementary Figure S5). The compound exhibited UV absorption at 240, 285, and 355 nm. The analysis of ^1^H and 2D HSQC (Heteronuclear single quantum coherence spectroscopy) and HMBC (Heteronuclear Multiple Bond Correlation) spectra revealed the presence of 25 carbon atoms, 12 quaternary carbons, five methine, six methylene, two methyl carbons and two *ortho*-coupled aromatic protons at δ_H_ 7.84 (dd, 8 Hz, 0.6 Hz, H-10) and δ_H_ 7.58 (d, 8 Hz, H-11) which indicate a tetrasubstituted aromatic ring. Key correlations in the HMBC spectrum from H-11 to C-7, C-7a, C-8, C-9, C-12, from H-6 to C-6a, C-12a, C-7, C-12, from H-5 to C-4a, C-12b, C-6a and from H-4 to C-4a, C-12b revealed the presence of the well-known aquayamycin-type core structure (ring A–D) substituted at position C-9 (Figure 4A; Supplementary Table S5; Supplementary Figures S6–S10). However, ring B is found to be fully saturated, which is only known for a few cases, e.g., moromycins A and B, grecocyclines, and N05WA963 A–C [19,20,53]. The ^13^C values, ranging from 70–80 ppm for C-4a, C-6a, C-12a, and C-12b indicated the presence of hydroxy groups whereas C-5 (δ_C_ 30.0, δ_H_ 1.65 and 2.21) and C-6 (δ_C_ 26.0, δ_H_ 2.18 and 2.47) were identified as CH_2_ groups. As known for the aquayamycin compounds, ring A bears a hydroxy and methyl group at C-3. The absolute configuration at this position has been determined as *R* in several previously isolated angucyclines with a similar biosynthetic origin, e.g., in urdamycin or saquayamycin [11,54]. Therefore, the configuration in baikalomycins A as well as in B is likely to be the same. In addition, one anomeric proton at δ_H_ 4.77 (δ_C_ 74.0), one methine proton at δ_H_ 3.22 (δ_C_ 72.5), two methylene signals at δ_H_ 1.43/2.20 (δ_C_ 33.0) and δ_H_ 1.63/2.11 (δ_C_ 33.5), and one methyl signal at δ_H_ 1.32 (δ_C_ 18.5) confirm the presence of a 2,3,6-tridesoxy-hexose unit. As known for other aquayamycin natural products, the sugar was attached to the aglycone at C-9, which was supported by HMBC correlations from H-1′ to C-8, C-9, C-10, as well as from H-2′ to C-9 (Figure 4A). Due to the large coupling constants of H-1’ (11 Hz), H-4′ (11, 9 Hz), and H-5′ (9 Hz), the respective protons must be in axial positions and therefore the sugar should be β-amicetose. The respective ROESY (Rotating frame Overhause Effect Spectroscopy) crosspeaks provided further proof for the given structure (Figure 4B). We assume that the sugar is *D* configured, as only the β-d-form of amicetose has been found among the C-glycosides in aquayamycin-like compounds so far [9]. Due to the fact that all substances could only be isolated in very small quantities and should still be subjected to biologic testing, a hydrolysis of the glycosides to determine the absolute configuration of the sugar components by means of optical rotation was not possible.

Baikalomycin B (**4**) was isolated as a pale-yellow solid (purity > 85 mol% according to ^1^H NMR, Supplementary Figure S11) with the molecular formula C_31_H_40_O_13_ and *m/z* 601.2332 [*M*-H_2_O-H]^−^ (calc. *m*/*z* 601.2285 [*M*-H_2_O-H]^−^), suggesting 12 degrees of unsaturation (Figure 3B; Supplementary Figure S5). Similar to (**3**), this compound showed absorption at 240, 285, and 355 nm. NMR data of ^1^H and ^13^C largely resembles the data of (**3**), however, with one major difference. The presence of an additional anomeric proton at δ_H_ 4.80 (δ_C_ 99.5), together with one methine proton at δ_H_ 3.15, two methylene groups at δ_H_ 1.76/1.84 and δ_H_ 1.74/1.78, and one methyl group at δ_H_ 1.18 indicate the presence of a second 2,3,6-tridesoxy-hexose moiety, which is *O*-glycosidically linked to the first sugar. HMBC correlations from H-1′′ to C-4′ confirm the connectivity of both sugar units (Figure 4B; Supplementary Table S5; Supplementary Figures S11–S15). The second sugar moiety was as well identified as amicetose due to the large diaxial coupling J_H4′′H5′′_ 9 Hz. Although the small coupling constant of H-1′′ (J_HH_ = 2.5 Hz) indicates the α-anomer in this case. Most probably, the α-amicetose is *L* configured as it was found for most of the terminal, O-glycosidically bound sugars in aquayamycin-like natural products [9].

Baikalomycin C (**5**) was obtained as a yellow solid (purity > 80 mol% according to ^1^H NMR, Supplementary Figure S16) and showed *m*/*z* 579.1887 [*M*-H]^−^ (calc. *m*/*z* 579.187184 [*M*-H]^−^) that corresponds to molecular formula C_31_H_32_O_11_ indicating 16 degrees of unsaturation (Figure 3B; Supplementary Figure S5). The UV absorption at 228, 258, 294, and 441 nm suggests an increased conjugated system compared to (**3**) and (**4**). Analysis of ^1^H NMR, HSQC, and HMBC spectra revealed an anthraquinone core formed by ring B, C, and D (Figure 4B; Supplementary Table S5; Supplementary Figures S16–S21). This finding was concluded from the *ortho*-coupled aromatic proton signals at δ_H_ 7.91 (d, 8 Hz, H-10) and δ_H_ 7.86 (d, 8 Hz, H-11) and HMBC correlations from H-10 to C-8, C-11a and from H-11 to C-7a, C-9, and C-12. A second *ortho*-coupled pair of protons at δ_H_ 7.76 (d, 8 Hz, H-5) and δ_H_ 7.80 (d, 8 Hz, H-6) confirm the presence of another aromatic ring. HMBC correlations from H-6 to C-12a and C-7 and from H-5 to C6a establish the connection of this ring to the quinone ring C. In contrast to (**3**) and (**4**), the former ring A was opened between C-1 and C-12b leading to a phenolic hydroxyl group at C-12b (δ_C_ 162.56) and a 3-hydroxy-3-methyl butanoic acid side chain attached to C-4a (Figure 3B). Similar to baikalomycin A and B, the C-glycosidic sugar attached to the aglycone at C-9 was identified as β-d-amicetose due to comparable chemical shift values and coupling constants for H-1′, H-4′ and H-5′ (Supplementary Table S5). An additional anomeric signal at δ_H_ 5.40 and δ_C_ 96.21 indicates a second O-glycosidic bonded sugar which is supported by corresponding HMBC correlations from H-1′′ to C-4′ (Supplementary Table S5). This sugar consists of two olefinic protons δ_H_ 6.98 (H-2′′), 6.05 (H-3′′), one methine proton δ_H_ 4.61, a methyl group at δ_H_ 1.32 and a carbonyl signal at δ_C_ 198.82 (C-4′′) and was thus identified as aculose. The small coupling constant of H-1′′ (3.5 Hz) is consistent with the α-anomer (Supplementary Table S5). Until today, only α-l-aculose was found in aquayamycin-type natural products, which let us assume that we have the sugar moiety [9]. The aglycone of baikalomycin C is the same as in amicenomycin B and himalomycin A [22,25]. Thus, the absolute configuration at C-3 can be assigned as *R*.

Despite the similarity to aquayamycin-type compounds, baikalomycins A and B possess an uncommon aglycone (hydroxy groups at C-6a and C-12a) that has been found in only one other angucycline, namely a derivative of PD 116198 isolated from *S. phaeochromogenes* WP 3688 [7]. As opposed to this, several angucyclinones and glycosylated angucyclines with epoxide function at these positions are known, including simocyclinones and grecocycline A [53,55]. Compounds with one hydroxyl group at C-6a or C-12a, like panglimycins A-B, saccharothrixmicine B, and kiamycin are also described [56,57,58]. The standalone case, however, is grecocycline B with a thiol group at C-6a and hydroxyl group at C-12a. Importantly, we were not able to find tri- and tetra-saccharides of baikalomycins in the extracts of *Streptomyces* sp. IB201691-2A.

The stereochemistry of the chiral centers in ring B of baikalomycins A and B remains uncertain. The spatial positions of the hydroxy groups at C-4a and C-12b were concluded to be *cis* as it has been described for all comparable angucyclines beforehand. However, the chiral centers C-6a and C-12a of the new aglycone could not be elucidated due to the lack of reliable signals for the hydroxyl protons even in DMSO-d6. Therefore, respective correlations involving the hydroxyl protons could not be found in the ROESY spectra.

### 3.4. Biological Activities of Baikalomycins

Rabelomycins and baikalomycins were tested for antibacterial and anticancer activities. The compounds **1**, **2,** and **5** showed moderate and weak activity against *Staphylococcus carnosus* DSMZ 20501 and *Mycobacterium smegmatis* DSMZ 43286 (Table 1). Also, **1**, **2,** and **4** were moderately active against *Erwinia persicina*.

Anticancer activities were determined against the human cancer cell lines A549 (lung carcinoma), Huh7.5 (hepatocellular carcinoma), MCF7 (breast adenocarcinoma), and SW620 (colorectal adenocarcinoma) by MTT assay [59]. Baikalomycins A and B showed moderate to weak effects on A549 and MCF7 cell viability (Table 2). The compounds **1**, **2**, and **5** exerted a more potent activity with IC_50_ values in the low micromolar range on all four cell lines (Table 2, Supplementary Figure S22A).

A549 cells were chosen to further analyze the potential antiproliferative actions in an impedance-based assay. Antiproliferative effects correlated with activities observed in A549 cells in the MTT assay: while **3**, **4,** and **5** showed no antiproliferative effects (data not shown), **1** and, to a greater extent, **2** reduced cell proliferation in concentrations showing no effect in the MTT assays (cell viability > 80% after 48 h treatment) (Supplementary Figure S22B).

### 3.5. Streptomyces sp. IB201691-2A Genome Sequencing and Analysis

The genome of *Streptomyces* sp. IB201691-2A has been sequenced and assembled into 109 contigs with an overall size of 11,410,308 bp (including 61,648 undefined nucleotides). The G+C content was found to be 70% that is typical for streptomycetes. The largest contig is 1155 kbp. The genome of *Streptomyces* sp. IB201691-2A consists of a single chromosome, based on the sequence coverage, and contains 10,023 predicted CDSs, 5 rRNA gene clusters, 86 tRNA, and one transfer-messenger RNA genes. AntiSMASH analysis revealed the presence of 38 gene clusters potentially involved in secondary metabolites biosynthesis (Supplementary Table S6). The genome is enriched with the genes for siderophores production, including those typical for actinobacteria desferrioxamine and two aerobactin-like siderophores biosynthetic gene clusters, as well as a more unusual scabichelin biosynthetic gene cluster, originally found in *S. scabies* [60]. The enrichment of *Streptomyces* sp. IB201691-2A with siderophores encoding gene clusters is not unusual, since the iron content of the Lake Baikal water is relatively low [61]. Biosynthetic products could also be clearly predicted for three terpene gene clusters, such as hopene (cluster 7), albaflavenone (cluster 13), and earthy-musty odor-causing 2-methylisoborneol (cluster 22) [62,63,64].

### 3.6. Identification of Baikalomycins Biosynthetic Gene Cluster

**Figure 5:**
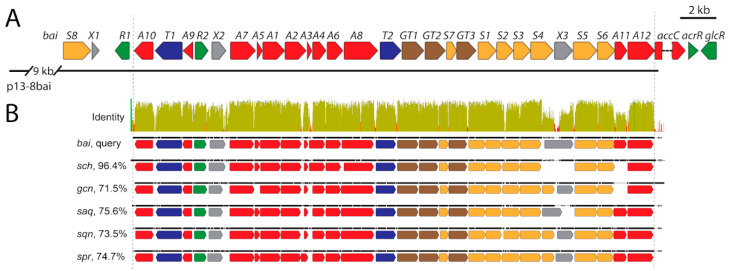
(**A**) Genetic organization of baikalomycin biosynthetic gene cluster (*bai*) from *Streptomyces* sp. IB 201691-2A. Region cloned in p13-8bai is shown below; (**B**) alignment of gene clusters responsible for biosynthesis of saquayamycin-type angucyclines from different actinobacteria. Mean pairwise identity over all pairs in the column: green—100% identity; green–brown—at least 30% and under 100% identity; red—below 30% identity. *bai*—baikalomycin gene cluster from *Streptomyces* sp. IB201691-2A; *sch*—Sch47554/47555 gene cluster from *Streptomyces* sp. SCC-2136; *gcn*—grincamycin gene cluster from *S. lusitanus* SCSIO LR32; *saq*—saquayamycin gene cluster from *S. nodosus* ATCC4899; *sqn*—saquayamycin gene cluster from *Streptomyces* sp. KY40-1; *spr*—saprolomycin gene cluster from *Streptomyces* sp. TK08046.

The genome of *Streptomyces* sp. IB201691-2A contains only one gene cluster encoding type II PKS and oligosaccharides biosynthesis enzymes that could be putatively involved in biosynthesis of baikalomycins (Figure 5A; Supplementary Table S6). This cluster, further designated as the *bai* gene cluster, is predicted to be 34 kbp in length and is located at the 3′ edge of scaffold00031. It encodes 27 complete and 1 incomplete CDSs. The BLAST analysis of the incomplete open reading frame (ORF) revealed that it is coding for putative acyl-CoA carboxylase with the closest homologue being SchP1 from Sch47554/47555 biosynthesis [29].

The search within the genome of *Streptomyces* sp. IB201691-2A identified a missing part of the acyl-CoA carboxylase gene at the 5′ edge of scaffold00038 (Supplementary Figure S23). We performed multiple sequence alignment of *bai*, *sch* (Sch47554/47555 from *Streptomyces* sp. SCC-2136), *saq* (saquayamycin from *S. nodosus* ATCC4899), *sqn* (saquayamycin from *Streptomyces* sp. KY40-1), *spr* (saprolomycin from *Streptomyces* sp. TK08046) and *gcn* (grincamycin from *S. lusitanus* SCSIO LR32) gene clusters (Figure 5B). This allowed determining the core of the *bai* cluster defined by genes *baiA10* encoding putative NADP-oxidase and *baiA12* methylmalonyl-CoA carboxylase. The genes within this region are present in all six closely related type II PKS-encoding clusters. The core of the *bai* gene cluster shares a 96.4% nucleotide sequence identity with the s*ch* cluster in pairwise alignment (Figure 5B; Supplementary Figure S23). At the same time, this number is lower for the other four related clusters. The high degree of similarity shows a close evolutional relationship between the mentioned biosynthetic genes that is reflected in the high similarity of produced compounds (Supplementary Figure S24).

#### 3.6.1. Genes Putatively Involved in Biosynthesis of Aglycone Core 

We attempted to predict the function of Bai enzymes in the assembly of baikalomycins. Genes *baiA1*, *baiA2,* and *baiA3* show high sequence similarity to *saqA*, *B*, *C*, *sqn H*, *I*, *J* and *SchP*6, 7, 8, encoding components of the “minimal PKS” complex (Supplementary Table S7): BaiA1 ketoacyl synthase α, BaiA2 ketoacyl synthase β (chain length factor CLF), and BaiA3 acyl carrier protein (ACP). These enzymes catalyze the synthesis of the nascent decaketide chain by repetitive condensation of one acetyl-CoA and nine malonyl-CoA units. BaiA4, showed high similarity to SchP5 (98%) and UrdD (86%, urdamycin ketoreductase from *S. fradiae* Tü2717) ketoreductases, responsible for the first reduction of the nascent polyketide chain at the C-9 position [29,65]. Two genes *baiA5*/*A6* code for putative cyclases/aromatases performing cyclization of the polyketide chain into the benz[a]anthracene structure. BaiA5/A6 resembles SchP4/P9 (99%/99%) and UrdF/L (73%/87%). *baiA7* encodes a close homologue of SrpB (77%) and UrdE (78%) proteins. UrdE is an oxygenase catalyzing hydroxylation of an angucycline aglycone at the C-12 position [66]. Lastly, *bai*A8 is encodes a second oxygenase enzyme with high identity to UrdM (85%) and SprI (88%) and seems to be involved in the hydroxylation in C-4a and C-12b [67]. Additionally, baikalomycins A and B bear hydroxy groups at positions C-6a and C-12a and lack C-5/C-6 double bond. A complete non-aromatic ring B has been rarely found in angucycline structures and thus there is no experimental data on how it is formed. It can be only assumed that hydroxylation of C-6a/C-12a and reduction of the double bond is catalyzed by oxygenase-reductase BaiA7, as it was hypothesized for kiamycin biosynthesis [68]. However, another scenario cannot be excluded. Two genes on the left edge of the *bai* cluster, *baiA9* and *baiA10*, code for putative flavin-oxidoreductase and NADPH-dependent reductase, respectively. The latter enzyme might provide the activity needed to reduce the double bond at C-5/C-6, when the former can be involved in processing of keto groups at C-6a/C-12a. Lastly, *baiA11* codes for putative 4′-phosphopantetheinyl transferase, possibly involved in activation of ACP, and *baiA12* encodes putative methylmalonyl-CoA carboxyltransferase, that might participate in precursors supply.

#### 3.6.2. Genes Putatively Involved in Deoxysugars Biosynthesis and Attachment

Two deoxysugars are present in the structure of baikalomycins: amicetose, in d- and l-configuration, and l-aculose. The *bai* gene cluster contains ten genes predicted to be involved in the biosynthesis of deoxysugars. Four enzymes, required for biosynthesis of the common precursor NDP-4-keto-2,6-dideoxy-d-glucose, are encoded by *baiS1* (glucose-1-phosphate thymidylyltransferase), *baiS2* (dTDP-glucose 4,6-dehydratase), *baiS5* (NDP-hexose 2,3-dehydratase), and *baiS6* (glucose-fructose oxidoreductase) (Figure 5A; Supplementary Table S7). Subsequently, the generation of d-amicetose is accomplished through the action of BaiS4 (dTDP-4-amino-4,6-dideoxy-d-glucose transaminase) and BaiS3 (dTDP-6-deoxy-l-talose-4-dehydrogenase). Lastly, epimerization at C-5′ would lead to formation of l-amicetose. BaiS7 has a high homology to UrdZ1 (71%) dTDP-4- dehydrorhamnose-3-epimerase.

The biosynthetic steps in the l-aculose formation were previously described for aclacinomycins and grincamycins [27,69]. In both cases l-aculose derives from rhodinose by the action of flavin-dependent oxidoreductases AknOx and GcnQ. Catalysis is performed in two steps: first, rhodinose is converted to cinerulose by oxidation at the C-4” position, followed by dehydrogenation to form double bond between C2” and C3” [69]. Unlike in the AknOx performed reaction, in the case of GcnQ no cinerulose intermediate has been observed [27]. Based on similarity with GcnQ and AknOx, it can be assumed that the flavin-dependent oxidoreductases SprY and SqnQ convert rhodinose to acculose in the case of saprolmycins and saquayamycins biosynthesis [28,31]. Corresponding genes are located at the 3′ edge of respective gene clusters downstream of the putative methylmalonyl-CoA carboxylase gene. In both, the *sch* and *bai* clusters, a gene encoding flavin-dependent oxidoreductase is missing in this region (Figure 5A). A BLAST search against *Streptomyces* sp. IB201691-2A genome using AknOx and GcnQ sequences as query resulted in one positive hit with high amino acid sequence identity (57.3% and 67.9%, respectively). The corresponding gene, 91_A2_44060 (*baiS8*), is located just upstream of initially defined borders of the *bai* cluster (Figure 5A; Supplementary Table S7). The *baiS8* orthologue in *sch* cluster *schA26*, also encodes flavin-dependent oxidoreductase. This makes BaiS8 and SchA26 the best candidates for enzymes catalyzing the conversion of amicetose to aculose.

Three genes, *baiGT1*, *baiGT2,* and *baiGT3,* are predicted to be involved in glycosylation steps in the assembly of baikalomycins (Figure 5A; Supplementary Table S7). BaiGT3 shows high homology to the C-glycosyltransferases SchS7 (98%), SprGT3 (79%), and UrdGT2 (73%), which catalyze a transfer of the first sugar to the C-9 position of the aglycone. Two other enzymes, BaiGT1 and BaiGT2, are predicted to be *O*-glycosyltransferases based on their homology to SchS10 (97%) and SchS9 (98%), respectively [30].

#### 3.6.3. Genes Involved in Regulation, Resistance, and with Unknown Functions

Two genes within the *bai* cluster might be involved in the regulation of baikalomycins production and control of its transport (Figure 5A; Supplementary Table S7). The *baiR1* gene product shows similarity to the LndI (66% identity) transcriptional regulator controlling landomycin E biosynthesis [70]. *baiR2* codes for TetR family transcriptional regulators that are widely distributed in bacteria, including *Streptomyces* genera, and typically control the expression of antibiotics transporter genes [71]. Two genes, bai*T1* and *baiT2*, which encode proteins putatively participating in the transport of baikalomycins and strain self-resistance, are found within the *bai* gene cluster. The *baiT1* gene product showed similarity to a major facilitator superfamily of the DHA2 group, typically implicated in multidrug resistance [72]. *baiT2* codes for a protein with 65% identity to PgaJ, a putative transmembrane efflux protein from the gaudimycin biosynthesis pathway [73]. Three genes *baiX1, X2,* and *X3* code for conserved hypothetical proteins with orthologues in many actinobacteria genomes but without functions assigned.

### 3.7. Inactivation of Genes Encoding Glycosyltransferases in bai Cluster 

**Figure 6:**
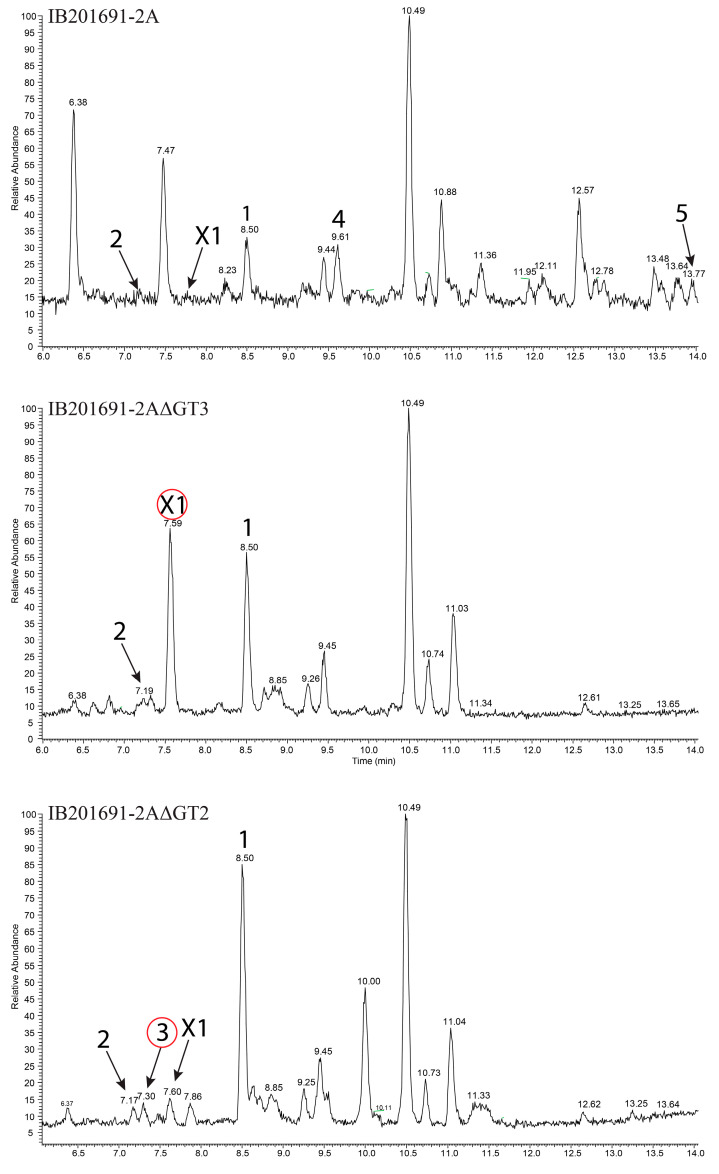
Analysis of LC-HRMS chromatograms of extracts of parental *Streptomyces* sp. IB201691-2A and mutant strains IB201691-2AΔGT3 and IB201691-2AΔGT2, lacking genes *baiGT3* and *baiGT2*, respectively, encoding baikalomycin glycosyltransferases. Compounds identified in the extracts: **1**—rabelomycin; **2**—5-hydroxy-rabelomycin; **3**—baikalomycin A; **4**—baikalomycin B; **5**—baikalomycin C; **X1**—baikalomycin aglycone.

In order to prove that the identified gene cluster is responsible for the production of baikalomycins and to investigate the specificity of glycosylation steps during biosynthesis we aimed to delete the genes *baiGT1*, *baiGT2,* and *baiGT3* within the chromosome of *Streptomyces* sp. IB201691-2A. For this, the suicide vector-based strategy was employed. However, the strain was found to be poorly genetically tractable. It took more than 20 attempts with each construct to obtain a few transconjugants. Unfortunately, we failed to introduce the *baiGT1* deletion construct into *Streptomyces* sp. IB201691-2A. In the case of two other genes, *baiGT2* and *baiGT3*, mutants were obtained by replacing the corresponding regions of the chromosome of *Streptomyces* sp. IB201691-2A with the spectinomycin resistance cassette. Mutant strains were cultivated in the production medium and extracted metabolites were analyzed by LC-MS (Figure 6). As expected, *Streptomyces* sp. IB201691-2AΔGT3, lacking *baiGT3*, accumulated rabelomycin (**1**) and 5-hydroxy- rabelomycin (**2**), but not glycosylated angucyclines (Figure 6). Furthermore, we found an additional peak (**X1**) in the extract of the mutant strain with RT of 7.56 and *m*/*z* 373.0945 [*M*-H2O-H]^−^ that corresponds to the mass calculated for aglycone of baikalomycins A and B (exact mass 392.11073, calculated *m*/*z* 373.092340 [*M*-H2O-H]^−^) (Figure 6; Supplementary Figure S25). This compound is also present in the extract of parental strain but in much smaller quantities.

Deletion of *baiGT2* abolished production of baikalomycins B and C. However, *Streptomyces* sp. IB201691-2AΔGT2 demonstrated increased accumulation of baikalomycin A that is barely detectable in the parental strain (Figure 6). These data lead us believe that BaiGT3 catalyzes the attachment of first amicetose at the C-9 of baikalomycins’ aglycone, followed by addition of a second amicetose by BaiGT2.

The second sugar might stay unprocessed or is converted to aculose by action of BaiS8, similar to how it occurs in the case of grincamycins and Sch 47554/47555 biosynthesis [27,30]. At the same time, the function of BaiGT1 remained unclear. In the case of Sch 47554/47555 biosynthesis SchS10, the BaiGT1 orthologue is responsible for introduction of a sugar (aculose or amicetose) at C-3 position. However, *Streptomyces* sp. IB201691-2A did not produce baikalomycin trisaccharides under multiple tested conditions. We were also not able to inactivate the *baiGT1* gene due to technical difficulties.

**Figure 7:**
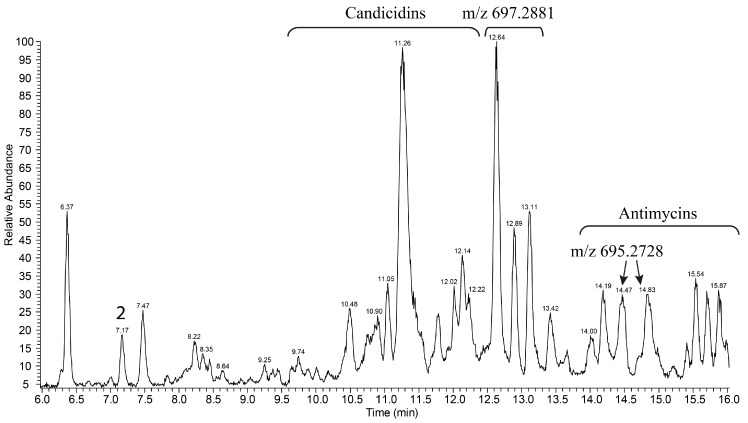
LC-MS chromatogram of extracts of *S. albus* J1074 carrying plasmid p8-13bai, with baikalomycin gene cluster from *Streptomyces* sp. IB201691-2A. Host exogenous compounds and new metabolites arisen from the expression of the *bai* gene cluster are highlighted.

We have cloned the entire *bai* gene cluster using transformation-associated recombination technique. As result, a plasmid p8-13bai, carrying 44.01 kb fragment of *Streptomyces* sp. IB201691-2A genomic DNA with the *bai* gene cluster, however, lacking *aacC* and other downstream genes, (Figure 5A) was expressed in *S. albus* J1074. As a result, the recombinant strain failed to produce baikalomycins A–C (Figure 7). At the same time, three peaks with *m/z* of 697.2882 [*M*-H]^−^ (RT 12.64, 12.89 and 13.11 min) and two peaks with *m*/*z* of 695.2734 [*M*-H]^−^ (RT 14.46 and 14.83 min) and characteristic for angucylines absorption spectrum could be found in the extract of *S. albus* J1074/p8-13bai (Figure 7, Supplementary Figure S26). These compounds are absent in the extract of *Streptomyces* sp. IB201691-2A. Based on the detected mass and UV spectra these metabolites are proposed to be baikalomycins trisaccharides closely related to Sch-47554/47555. MS2 data support this idea. All five new metabolites have typical angucycline type fragmentation pattern with the characteristic loss of one sugar, most probably at C-3 position (Supplementary Figure S27). Most plausible, that the variety of masses of the detected compounds originate from incomplete conversion of amicetose to aculose, but rather to cineruloses intermediate in one or both saccharides at C-3 and C-9 positions, as it was shown for grincamycins [27].

In the latter case, expression of the grincamycin biosynthesis genes in *S. coelicolor* yielded vineomycin A1 with two aculose moieties instead of cineruloses, present in grincamycins. It is obvious that the baikalomycins biosynthetic enzymes behave differently in the natural producer and the heterologous host. In fact, *Streptomyces* sp. IB201691-2A did not produce angucycline trisaccharides under any of tested cultivation conditions but accumulated large amounts of shunt product rabelomycin and its derivatives. Vice versa, *S. albus* carrying the *bai* gene cluster accumulated the angucycline trisaccharides. In conclusion, six gene clusters encoding the aquayamycin-type angucyclines biosynthesis have a high degree of sequence identity (Figure 5B) and, at the same time, are responsible for the production of a great variety of related, but still different, natural products. Such variety, obviously, cannot be deduced from the nucleotide sequence analysis and thus leaves high chances to discover new natural products from the highly similar biosynthetic gene clusters.

## Figures and Tables

**Table 1:** Activity tests of baikalomycins A–C (**3–5**), rabelomycin (**1**), and 5-hydroxy-rabelomycin (**2**).

Test Strain	MIC, μM				
	3	4	5	1	2
*Erwinia persicina* DSMZ 19328	>500	250	n.t.	31	125
*Pseudomonas putida* KT2440	>500	>500	>500	>500	>500
*Candida glabrata* DSMZ 11226	>500	>500	>500	>500	>500
*Staphylococcus carnosus* DSMZ 20501	>500	>500	62	62	125
*Mycobacteriaum smegmatis* DSMZ 43286	>500	>500	250	31	125

**Table 2:** IC_50_ values [µM] of baikalomycins A–C (**3–5**), rabelomycin (**1**), and 5-hydroxy-rabelomycin (**2**) ± SEM against human tumor cell lines, treated for 48 h.

Compound	A549	Huh7.5	MCF7	SW620
**3**	58.51 ± 5.15	inactive	53.19 ± 3.36	inactive
**4**	46.26 ± 0.52	inactive	inactive	inactive
**5**	42.43 ± 3.71	7.62 ± 0.47	13.35 ± 1.33	3.87 ± 0.69
**1**	9.78 ± 0.49	7.21 ± 0.70	21.94 ± 1.59	7.82 ± 0.40
**2**	9.11 ± 0.59	11.91 ± 2.94	27.39 ± 2.17	13.43 ± 0.72

## Supplementary Materials

The following are available online at https://www.mdpi.com/2076-2607/8/5/680/s1.
