# Supplementary material for: Baikalomycins A-C, New Aquayamycin-Type Angucyclines Isolated from Lake Baikal Derived Streptomyces sp. IB201691-2A
Source: Microorganisms. 2020 May 7;8(5):680. doi: 10.3390/microorganisms8050680 (PMC7284819; doi:10.3390/microorganisms8050680)
Supplement: Supplementary file 1 [file microorganisms-08-00680-s001.pdf]

Supporting Information

Baikalomycins A-C, New Aquayamycin-Type Angucyclines Isolated from Lake Baikal Derived *Streptomyces* sp. IB201691-2A

Irina Voitsekhovskaia [1,†,‡], Constanze Paulus [2,†], Charlotte Dahlem [3], Yuriy Rebets [4], Suvd Nadmid [4,§], Josef Zapp [3], Denis Axenov-Gribanov [1,5], Christian Rückert [6], Maxim Timofeyev [1,5], Jörn Kalinowski [6], Alexandra K. Kiemer [3] and Andriy Luzhetskyy [2,4,*]

[1] Institute of Biology, Irkutsk State University, 664003 Irkutsk, Russia; irina.voytsekhovskaya@gmail.com (I.V.); denis.axengri@gmail.com (D.A.-G.); m.a.timofeyev@gmail.com (M.T.)

[2] Helmholtz Institute for Pharmaceutical Research Saarland, 66123 Saarbrücken, Germany; Constanze.Paulus@helmholtz-hzi.de

[3] Pharmaceutical Biology, Saarland University, 66123 Saarbrücken, Germany; charlotte.dahlem@uni-saarland.de (C.D.); j.zapp@mx.uni-saarland.de (J.Z.); pharm.bio.kiemer@mx.uni-saarland.de (A.K.K.)

[4] Pharmaceutical Biotechnology, Saarland University, 66123 Saarbrücken, Germany; y.rebets@mx.uni-saarland.de (Y.R.); suvdn@yahoo.com (S.N.)

[5] Baikal Research Centre, 664003 Irkutsk, Russia

[6] Technology Platform Genomics, Center for Biotechnology (CeBiTec), Bielefeld University, 33615 Bielefeld, Germany, Christian.Rueckert@cebitec.uni-bielefeld.de (C.R.); joern@cebitec.uni-bielefeld.de (J.K.)

* Correspondence: a.luzhetskyy@mx.uni-saarland.de; Tel.: +49-681-302-70200

† These authors contributed equally to this work.

‡ Current address: Interfaculty Institute of Microbiology and Infection Medicine (IMIT), Microbiology/Biotechnology, Eberhard Karls University of Tübingen, 72074 Tübingen, Germany; irina.voitsekhovskaia@uni-tuebingen.de

§ Current address: School of Pharmacy, Mongolian National University of Medical Sciences, 14210 Ulaanbaatar, Mongolia

Received: 7 April 2020; Accepted: 5 May 2020; Published: date

Table S1. Primers used in this work.

Primer	Sequence 5'-3'
***bai* gene cluster cloning**	
91-2aTAR1-FNotI	atGCGGCCGCAGACGTGTGTACTTCGCATG
91-2aTAR1-RNheI	atGCTAGCTCTCGTGCGGGGAGTCGAT
91-2aCheck-FNotI	AGGTACGAGTCCGTCGGATA
91-2aTAR2-FNheI	atGCTAGCATCGGATGATGGAGCGTCAA
91-2aTAR2-RHindIII	atAAGCTTTCAACACCTGGATGGACGGA
91-2aCheck-RHindIII	GTTATGAACTGTGGGGCAG
pCLY10-FHindV	CTCTTCGGTGAACTTCGGG
pCLY10-RNotV	GTAGAGCTTCCGGCTCGTAT
**Disruption of glycosyltransferase genes**	
3L-FHindIII	AAGCTTGATCTGGGGCTCTCGATGTG
3L-REcoRV	GATATCTCACGAGTACGCGCATCTGA
4R-FEcoRV	GATATCCTCACCTTCGACAAGGCAGC
4R-RXbaI	TCTAGACGAGGTACATCACGAAGTCG
5L-FHindIII	AAGCTTCAAGATCTTCGGCAGGTACA
5L-REcoRV	GATATCCGAAGAGGATCCTCACAATG
6R-FEcoRV	GATATCGAACTCTCCGCAGAGATCGC
6R-RXbaI	TCTAGAGACACTGCCGAGCTGGATCA
CheckGT2F	CCCGTCGTTCCAGCTCGGTG
CheckGT2R	CGTGTCGTCCCGCATCGCCT
CheckGT3F	AGCTCGATCTGCCGTGGGAC
CheckGT3R	GCATCGAATAACTGAAGGGT
**16S rRNA**	
8F	AGAGTTTGATYMTGGCTCAG
1492R	TACGGYTACCTTGTTACGACTT

Table S2. *16S* rRNA gene sequence used for phylogenetic analysis.

Related strain	Accessory N of isolates in NCBI database	Identity, %	Query cover, %
Streptomyces ederensis NBRC 15410	NR_112457.1	99	97
Streptomyces phaeochromogenes NRRL B-1248	NR_116382.1	99	98
Streptomyces umbrinus NBRC 13091	NR_041113.1	99	97
Streptomyces aurantiacus LMG 19358	NR_114932.1	99	97
Streptomyces bottropensis ATCC 25435	NR_115571.2	98	100
Streptomyces glomeroaurantiacus NBRC 15418	NR_041436.1	99	95
Streptomyces avermitilis MA-4680	NR_074747.2	98	100
Streptomyces durmitorensis MS405	NR_043520.1	97	99
Rhodococcus phenolicus DSM 44812	NR_115082.1	88	99
Rhodococcus zopfii DSM 44108	NR_041775.1	88	99
Rhodococcus rhodochrous DSM 43241	NR_116689.1	89	99
Rhodococcus pyridinivorans PDB9	NR_025033.1	88	99
Rhodococcus marinonascens DSM 43752	NR_026183.1	88	99
Rhodococcus erythropolis N11	NR_037024.1	88	99
Rhodococcus coprophilus CUB 687	NR_029206.1	90	99

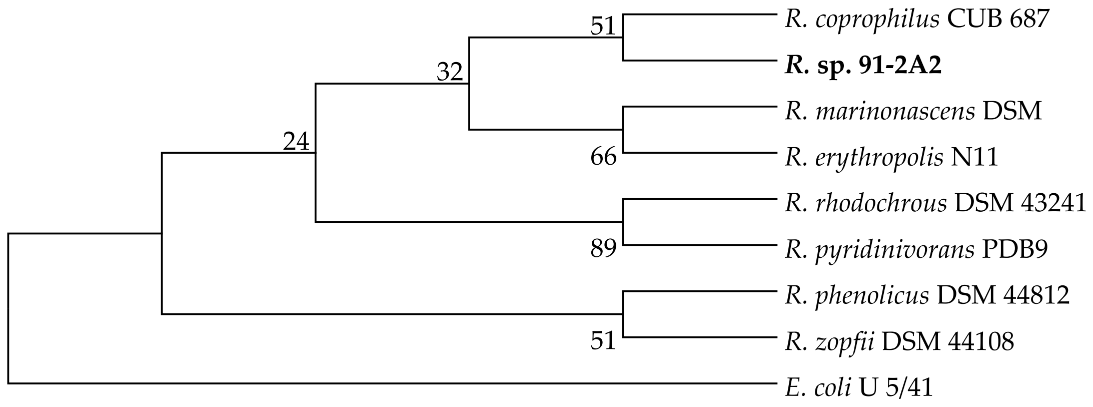

Figure S1. Taxonomic relation of *Rhodococcus* sp. IB201691-2A2 based on *16S* rRNA gene similarity. *Escherichia coli* U 5/41 was used as outgroup.

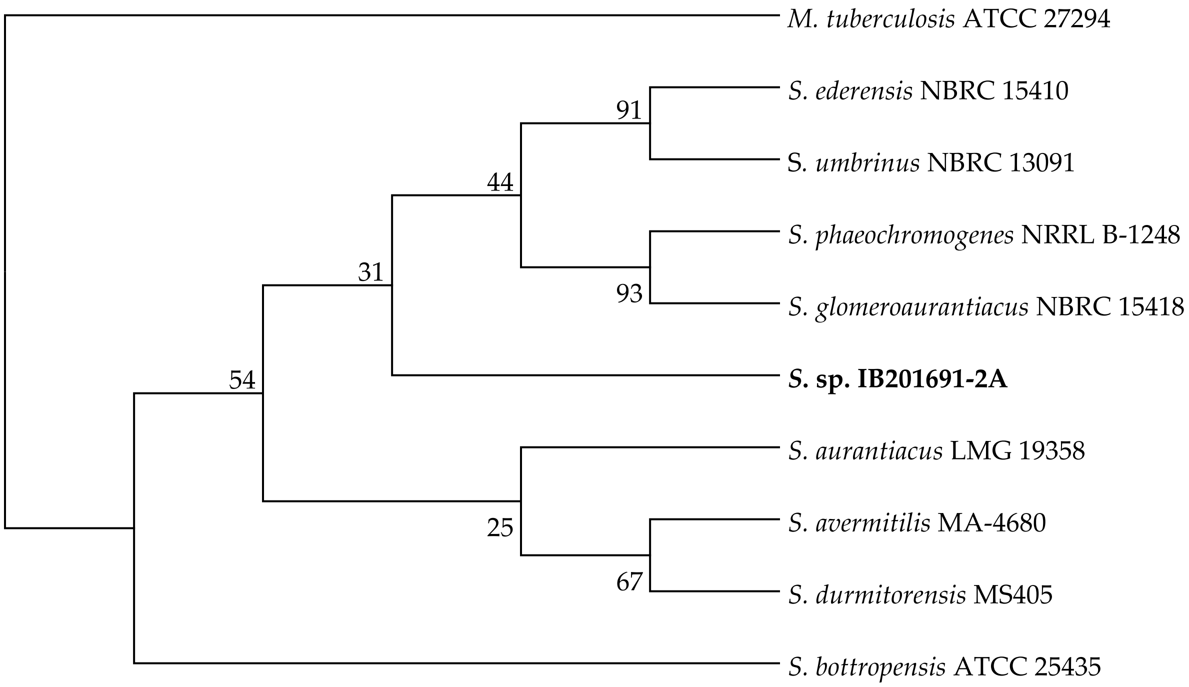

Figure S2. Taxonomic relation of *Streptomyce*s sp. IB201691-2A based on *16S* rRNA gene similarity. *Mycobacterium tuberculosis* ATCC 27294 was used as outgroup.

Table S3. Activity tests against bacterial and yeast test cultures of *Streptomyces* sp. IB201691-2A crude extracts from cultural liquid, cultivated in different media and conditions.

Temperature of cultivation	13°C					28°C					37°C			
Medium	*B.subtilis*	*P.putida*	*E.coli*	*S.cerevisiae*		*B.subtilis*	*P.putida*	*E.coli*	*S.cerevisiae*		*B.subtilis*	*P.putida*	*E.coli*	*S.cerevisiae*
Sm1	9	-	-	-		10	-	-	-		-	-	-	-
Sm17	9	-	-	-		8	-	-	<7		-	-	-	-
Sm12	<7	-	-	-		-	-	-	8		-	-	-	-
Sm20	-	-	-	-		-	-	-	-		-	-	-	-
Sm24	-	-	-	-		-	-	-	-		-	-	-	-
Sm25	-	-	-	-		-	-	-	-		-	-	-	-
Sm27Ac	<7	-	-	-		13	-	-	-		-	-	-	-
Sm27N	-	-	-	-		15	-	-	-		-	-	-	-
Sm27Al	-	-	-	-		-	-	-	-		-	-	-	-
R2	-	-	-	-		12	-	-	-		-	-	-	-
MM	9	-	-	-		10	-	-	-		-	-	-	-

* zones of inhibition in mm

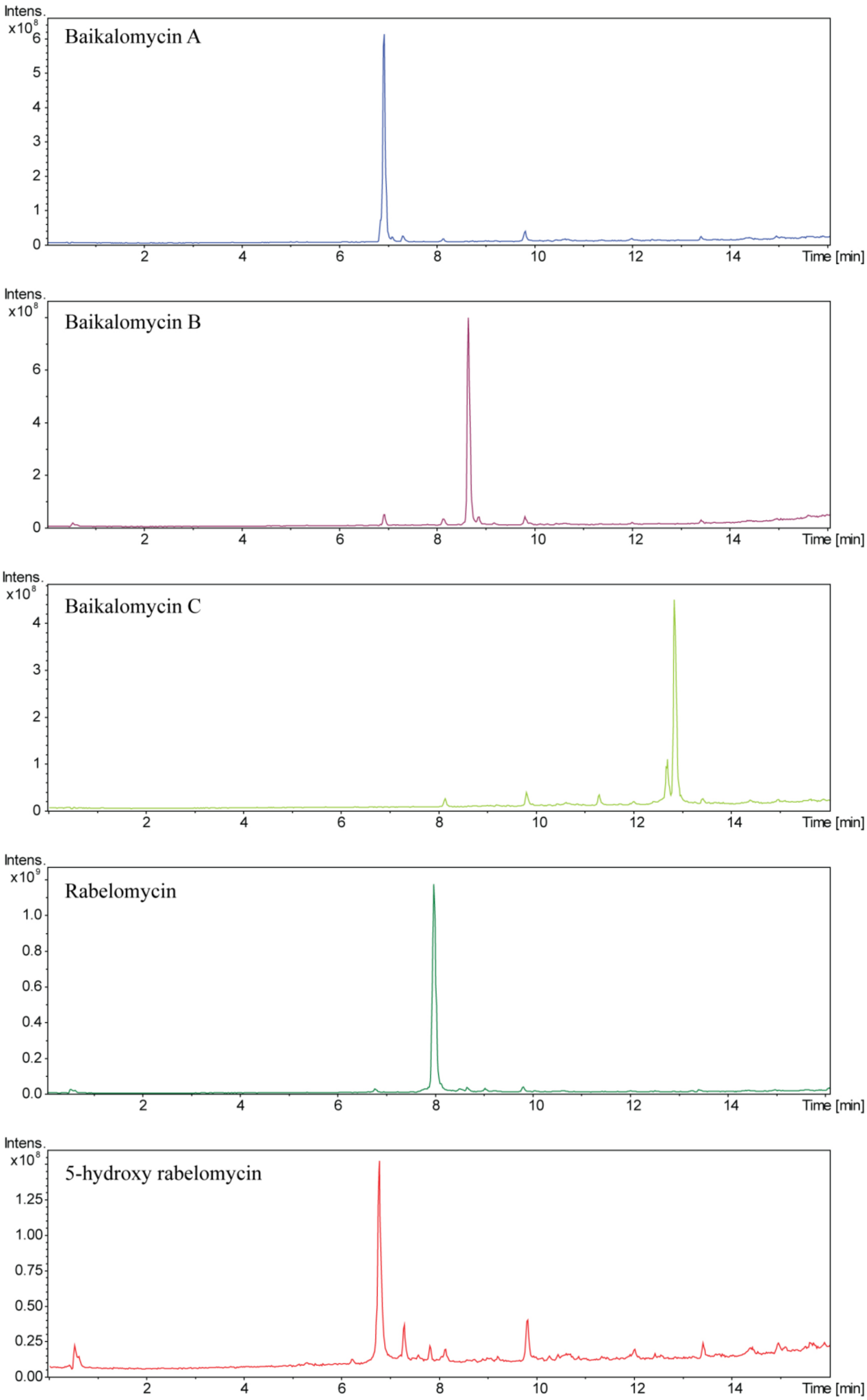

Figure S3. LC-MS chromatograms of pure compounds isolated in this work.

Purity of Baikalomycins A-C.

Despite MS and UV control during isolation, all ^1H NMR spectra of Baikalomycins A-C still showed signals for long-chain alkyl moieties (methyl triplets at 0.90 and broad methylene singlets at 1.28 ppm), which are typical for saturated fats. According to the integration of the methyl proton signals, their content is about 15 mol% (baikalomycin A and B) or 20 mol% (baikalomycin C). In baikalomycin A and B even smaller amounts of an unspecified compound (< 5 mol%) with double bond protons at 6.08 and 6.18 ppm, both d, could be detected. All in all, the purity of Baikalomycins can be stated as follows: Baikalomycin A and C: > 80%, Baikalomycin B: > 85%.

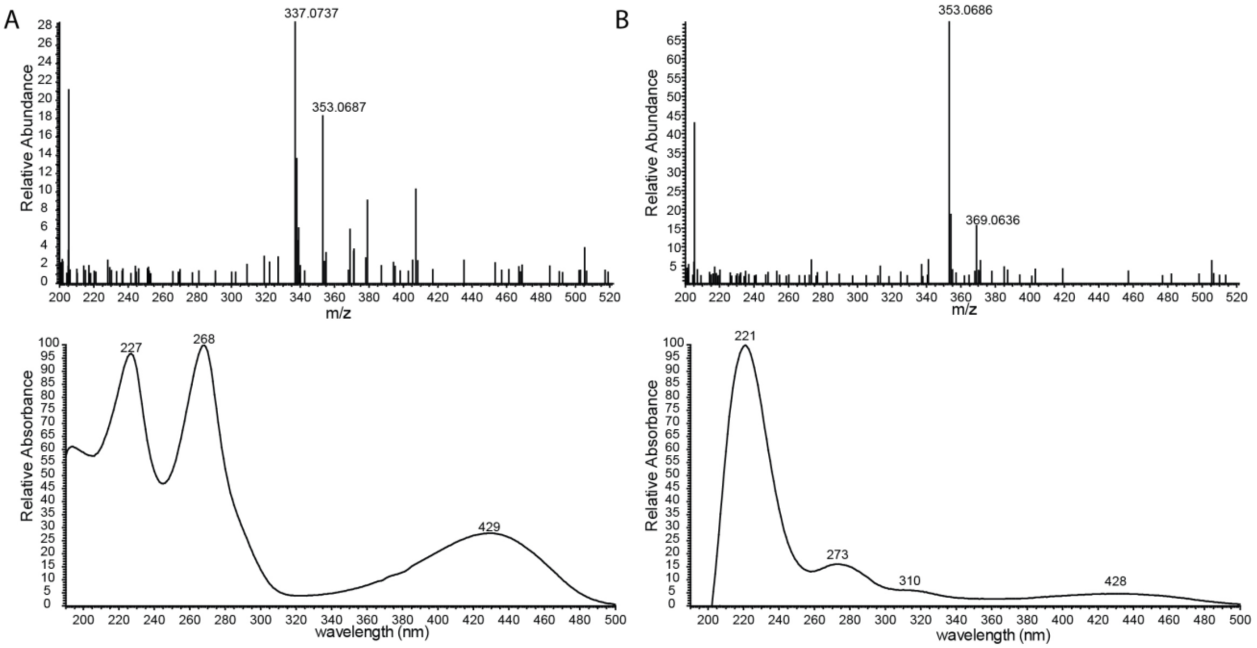

Figure S4. (A) MS and UV absorption spectra of rabelomycin (**1**) (RT of 8.0 min; observed m/z 337.0738 $[M-H]^-$, that corresponds to exact mass 338.081; calculated exact mass of rabelomycin 338.07904)**.** (B) MS and UV absorption spectra of 5-hydroxy-rabelomycin (**2**) (RT of 6.8 min, observed m/z 353.0687 $[M-H]^-$, that corresponds to exact mass 354.0759; calculated exact mass of 5-hydroxy-rabelomycin 354.07395).

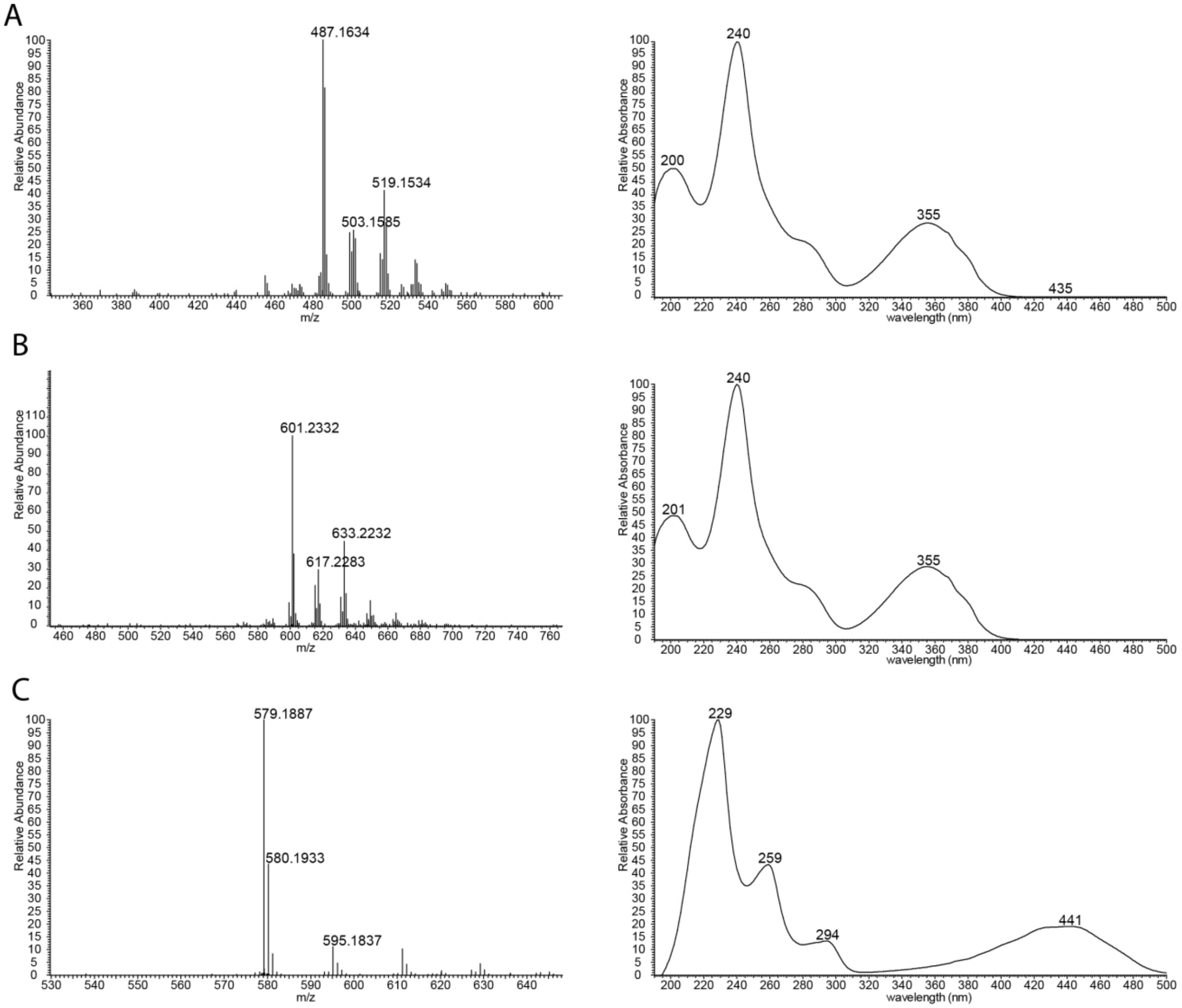

Figure S5. MS and UV absorption spectra of baikalomycins A (A), B (B) and C (C).

Table S4. NMR spectroscopic data for rabelomycin (1) and 5-hydroxy-rabelomycin (2) in DMSO-d_6.

	**(1)**		**(2)**	
Position	δ_C [a, b]	δ_H (J in Hz)	δ_C [a, b]	δ_H (J in Hz)
**1**	196.0, C	-	196.3, C	-
**2**	53.0, CH_2	2.92, d (14.4)<br>2.66, d (14.4)	52.6, CH_2	2.93<br>2.63
**3**	71.1, C	-	71.0, C	-
**4**	43.4, CH_2	3.10, d (16.8)<br>2.97, d (16.8)	37.5, CH_2	3.06<br>2.83
**4a**	152.0, C	-	147.5, C	-
**5**	121.4, CH	7.11, s	135.4, C	-
**6**	162.0, C	-	162.3, C	-
**6a**	116.6, C	-	115.9, C	-
**7**	191.1, C	-	191.2, C	-
**7a**	115.4, C	-	115.3, C	-
**8**	160.4, C	-	160.4, C	-
**9**	123.4, CH	7.32, d (8.3)	122.4, CH	7.30, d (8.5)
**10**	137.4, CH	7.76, t (8.3	136.7, CH	7.75, t (7.9)
**11**	118.5, CH	7.46, d (7.4)	117.9, CH	7.46, d (7.4)
**11a**	135.4, C	-	135.6, C	-
**12**	183.4, C	-	181.6, C	-
**12a**	137.6, C	-	135.4, C	-
**12b**	129.2, C	-	129.9, C	-
**13**	29.3, CH_3	1.30, s	29.9, CH_3	1.34, s

[a] = followed by multiplicity;
[b] = ^{13}C chemical shifts taken from 2D spectra HSQC/HMBC

Table S5. NMR spectroscopic data for baikalomycins A-C (3-5) in CD_3OD.

	**(3)**		**(4)**		**(5)**	
Position	δ_C [a, b]	δ_H (*J* in Hz)	δ_C [a, b]	δ_H (*J* in Hz)	δ_C [a]	δ_H (*J* in Hz)
**1**	207.0, C	-	207.0, C	-	179.18, C	-
**2**	49.0, CH_2	2.48 (m, 2H)	49.0, CH_2	2.49 (m, 2H)	47.56, CH_2	2.41 (d, 15)<br>2.36 (d, 15)
**3**	76.5, C	-	76.5, C	-	73.00, C	-
**4**	48.0, CH_2	2.38 (dd, 14.8, 2 Hz)<br>1.87 (d, 14.8 Hz)	48.0, CH_2	2.38 (dd, 14.8, 2 Hz)<br>1.87 (d, 14.8)	41.16, CH_2	3.05 (d, 13.3 Hz)<br>3.03 (d, 13.3 Hz)
**4a**	72.5, C	-	72.5, C	-	136.73, C	-
**5**	30.0, CH_2	2.21 (m)<br>1.65 (m)	30.0, CH_2	2.20 (m)<br>1.64 (m)	140.82, CH_2	7.76 (d, 8 Hz)
**6**	26.0, CH_2	2.47 (m)<br>2.18 (m)	26.0 CH_2	2.47 (m)<br>2.18 (m)	119.55, CH_2	7.80 (d, 8 Hz)
**6a**	78.0, C	-	78.5, C	-	133.01, C	-
**7**	201.0, C	-	201.0, C	-	189.65, C	-
**7a**	116.0, C	-	116.0, C	-	116.74, C	-
**8**	159.0, C	-	159.0, C	-	160.00, C	-
**9**	140.0, C	-	140.0, C	-	140.15, C	-
**10**	134.0 CH	7.84 (dd, 8, 0.6 Hz)	134.0, CH	7.85 (dd, 8, 0.6 Hz)	134.22, CH	7.91 (d, 8 Hz)
**11**	120.0, CH	7.58 (d, 8 Hz)	120.0, CH	7.58 (d, 8 Hz)	120.12, CH	7.86 (d, 8 Hz)
**11a**	133.0, C	-	133.0, C	-	133.37, C	-
**12**	193.0, C	-	193.0, C	-	189.50, C	-
**12a**	81.0, C	-	81.0, C	-	116.69, C	-
**12b**	79.5, C	-	79.5, C	-	162.56, C	-
**13**	25.0, CH_3	1.17 (s, 3H)	25.0, CH_3	1.17 (s, 3H)	27.36, CH_3	1.21 (s, 3H)
**1′**	74.0, CH	4.77 (dd, 11, 2 Hz)	74.0, CH	4.79 (dd, 11, 2 Hz)	74.47, CH	4.85 (dd, 11, 2 Hz)
**2′**	33.0, CH_2	2.20 (m)<br>1.43 (tdd, 13.5, 11, 3.7 Hz)	32.5, CH_2	2.23 (m)<br>1.44 (tdd, 13.5, 11, 4 Hz)	32.83, CH_2	2.27 (m)<br>1.52 (tdd, 13.5, 11, 4 Hz)
**3′**	33.5, CH_2	2.11 (ddt, 12.5, 4.5, 3.5 Hz)<br>1.63 (m)	32.5, CH_2	2.26 (m)<br>1.75 (m)	32.74, CH_2	2.36 (m)<br>1.82 (tdd, 13.5, 11, 4 Hz)
**4′**	72.5, CH	3.22 (ddd, 11, 9, 4.5 Hz)	81.0, CH	3.26 (ddd, 11, 9, 4.5 Hz)	82.06, CH	3.45 (ddd, 11, 9, 4.5 Hz)
**5′**	80.0, CH	3.37 (dq, 9, 6 Hz)	79.0, CH	3.48 (dq, 9 Hz, 6 Hz)	78.64, CH	3.54 (dq, 9, 6 Hz)
**6′**	18.5, CH_3	1.32 (d, 6 Hz)	18.0, CH_3	1.30 (d, 6 Hz)	18.81, CH_3	1.37 (d, 6 Hz)
**1′′**	-		99.5, CH	4.80* (d, 2.5 Hz)	96.21, CH	5.40 (d, 3.5 Hz)
**2′′**	-	-	31.0, CH_2	1.84 (m)<br>1.76 (m)	145.59, CH	6.98 (dd, 10, 3.5 Hz)
**3′′**	-	-	28.0, CH_2	1.78 (m)<br>1.74 (m)	127.64, CH	6.05 (d, 10 Hz)
**4′′**	-	-	72.5, CH	3.15 (m)	198.82, C	-
**5′′**	-	-	71.0, CH	3.70 (dq, 9, 6 Hz)	71.44, CH	4.61 (q, 6.8 Hz)
**6′′**	-	-	18.5, CH_3	1.18 (d, 6 Hz)	15.48, CH_3	1.32 (d, 6.8 Hz)

*taken from the ^1H NMR in DMSO-d_6. This signal was overlapped by HDO in CD_3OD spectrum

[a] = followed by multiplicity;

[b] = ^{13}C chemical shifts taken from 2D spectra HSQC/HMBC

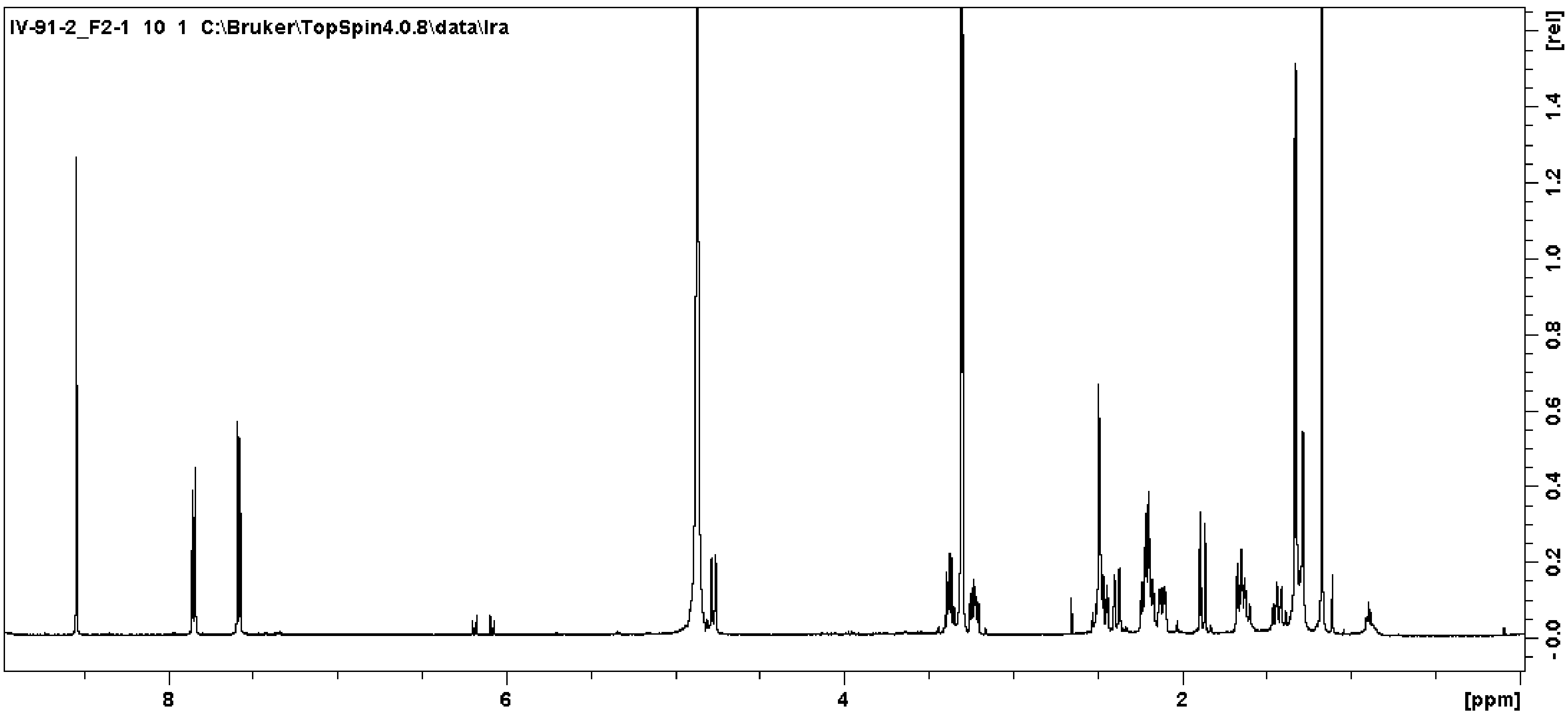

Figure S6. ^{1}H spectra of Baikalomycin A in CD_3OD.

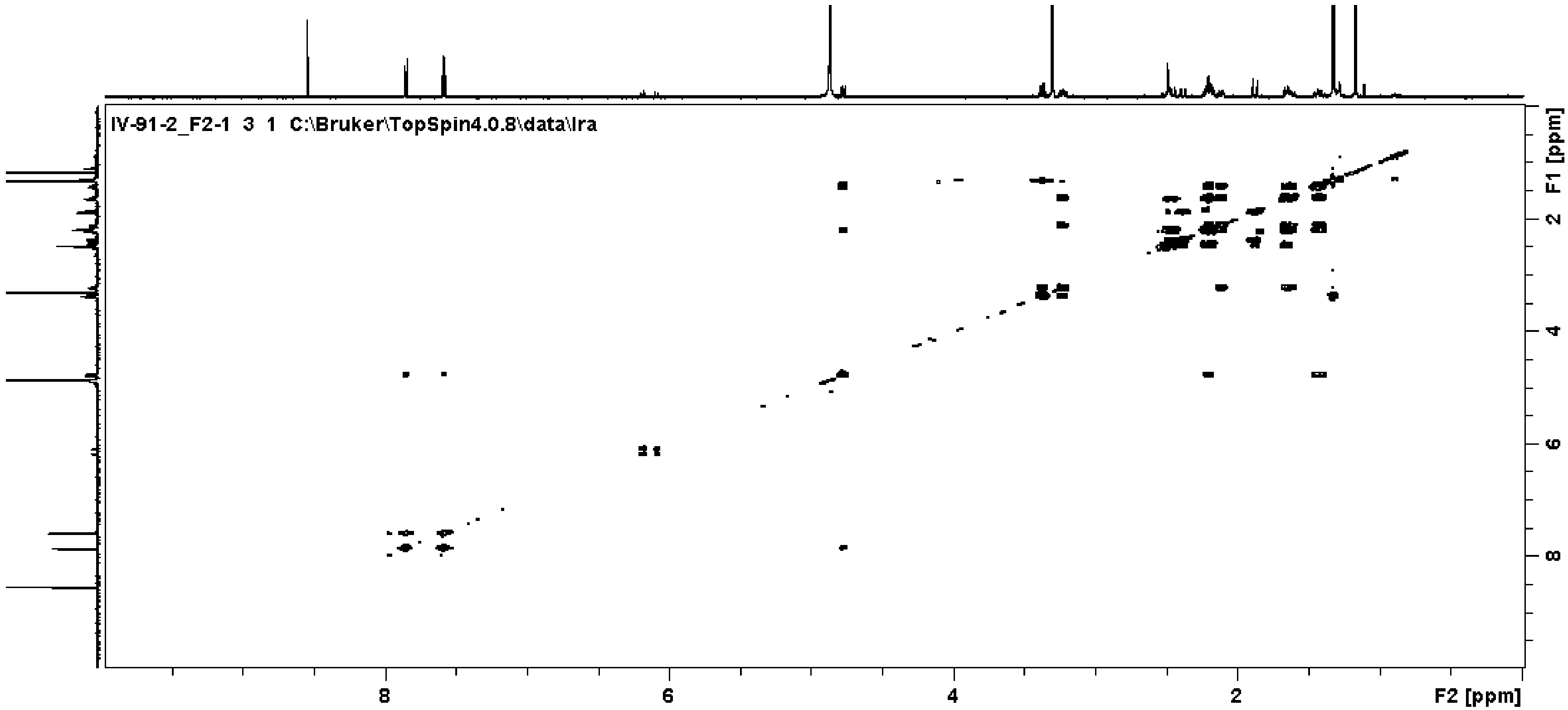

Figure S7. ^{1}H-^{1}H-Cosy spectra of Baikalomycin A in CD_3OD.

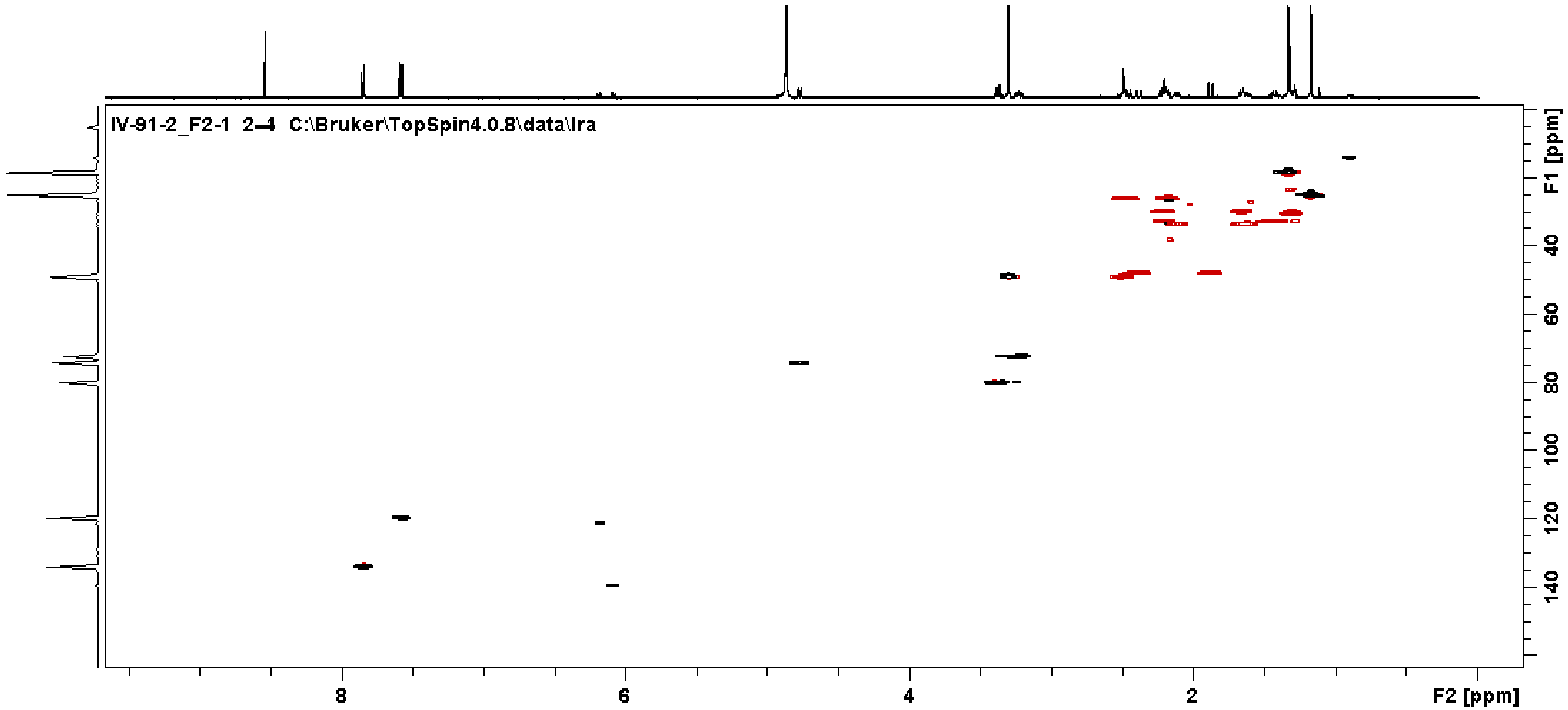

Figure S8. HSQC spectra of Baikalomycin A in CD_3OD.

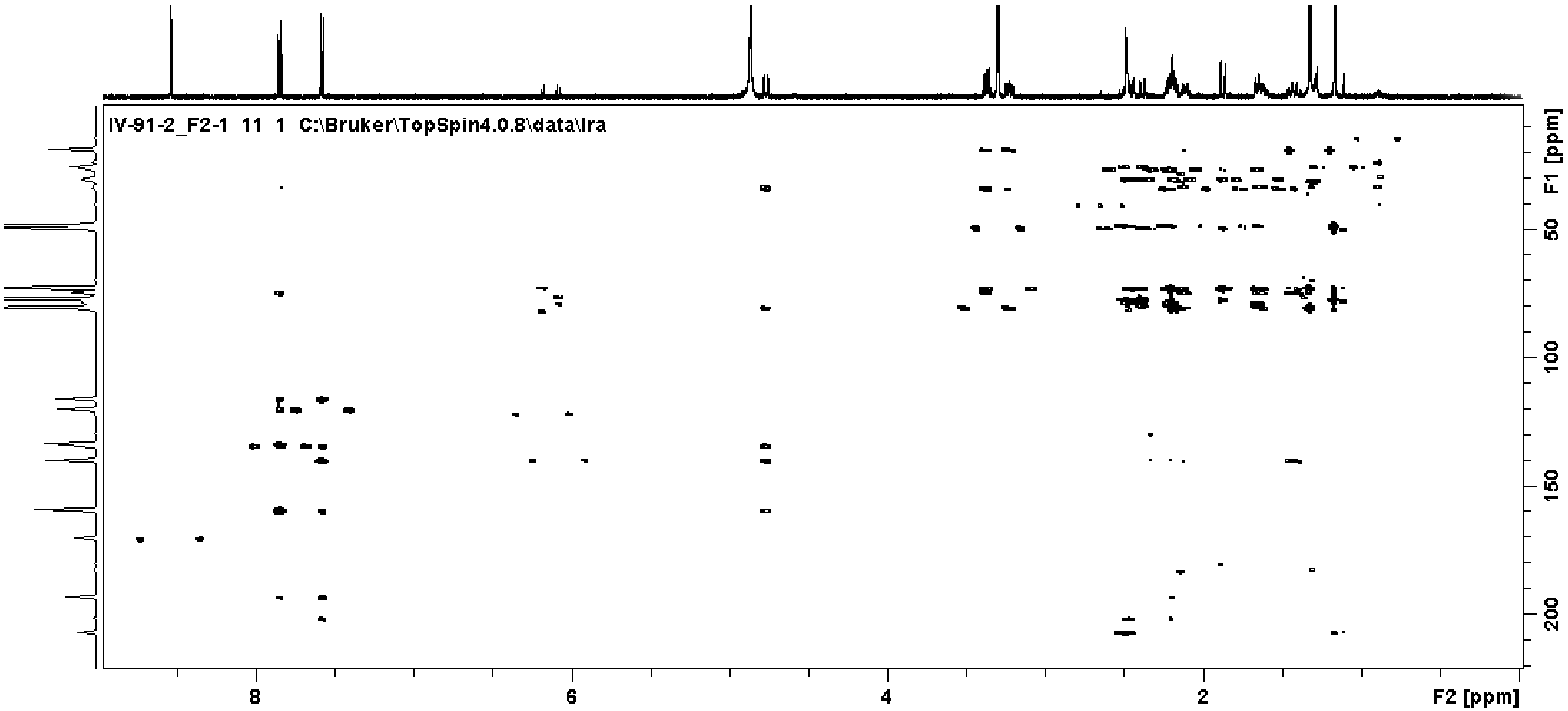

Figure S9. HMBC spectra of Baikalomycin A in CD_3OD.

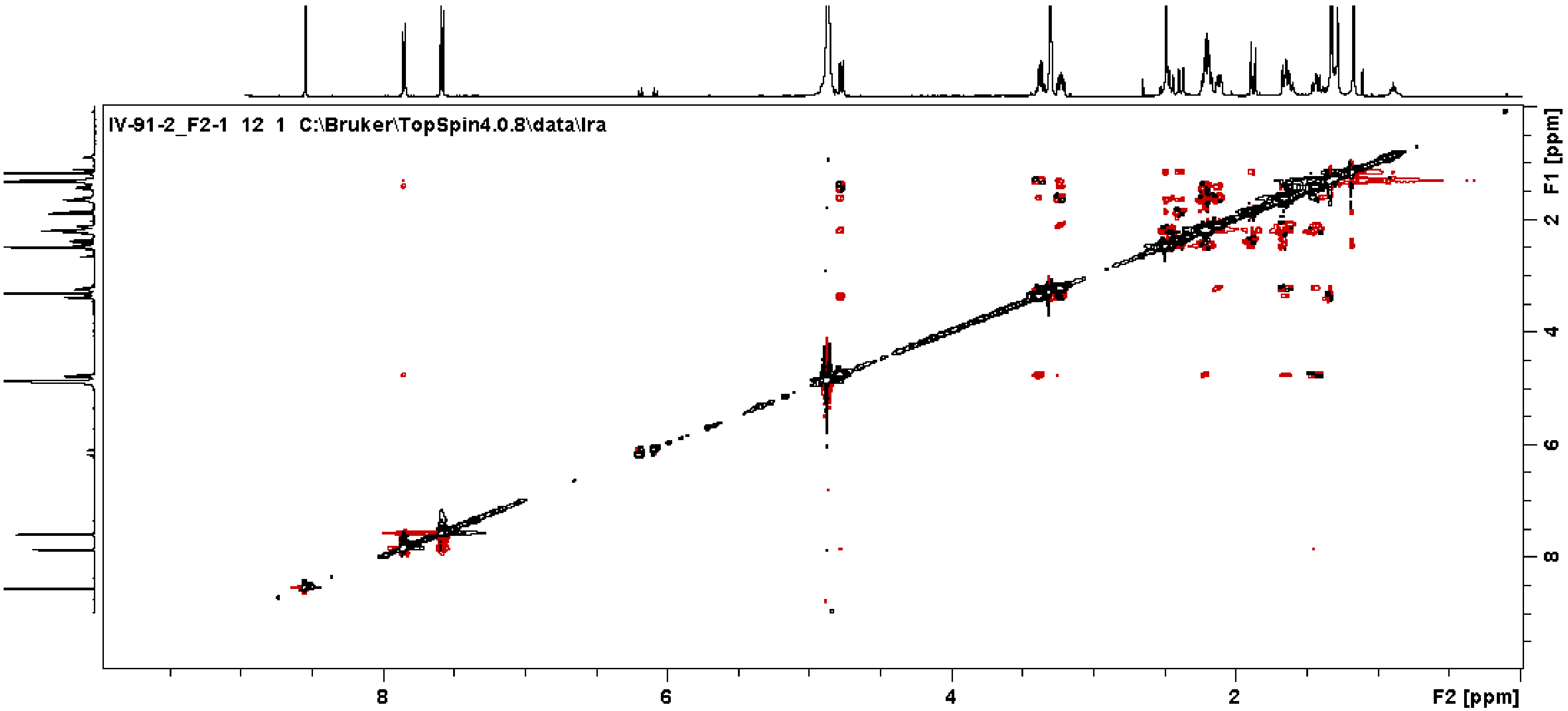

Figure S10. ROESY spectra of Baikalomycin A in CD_3OD.

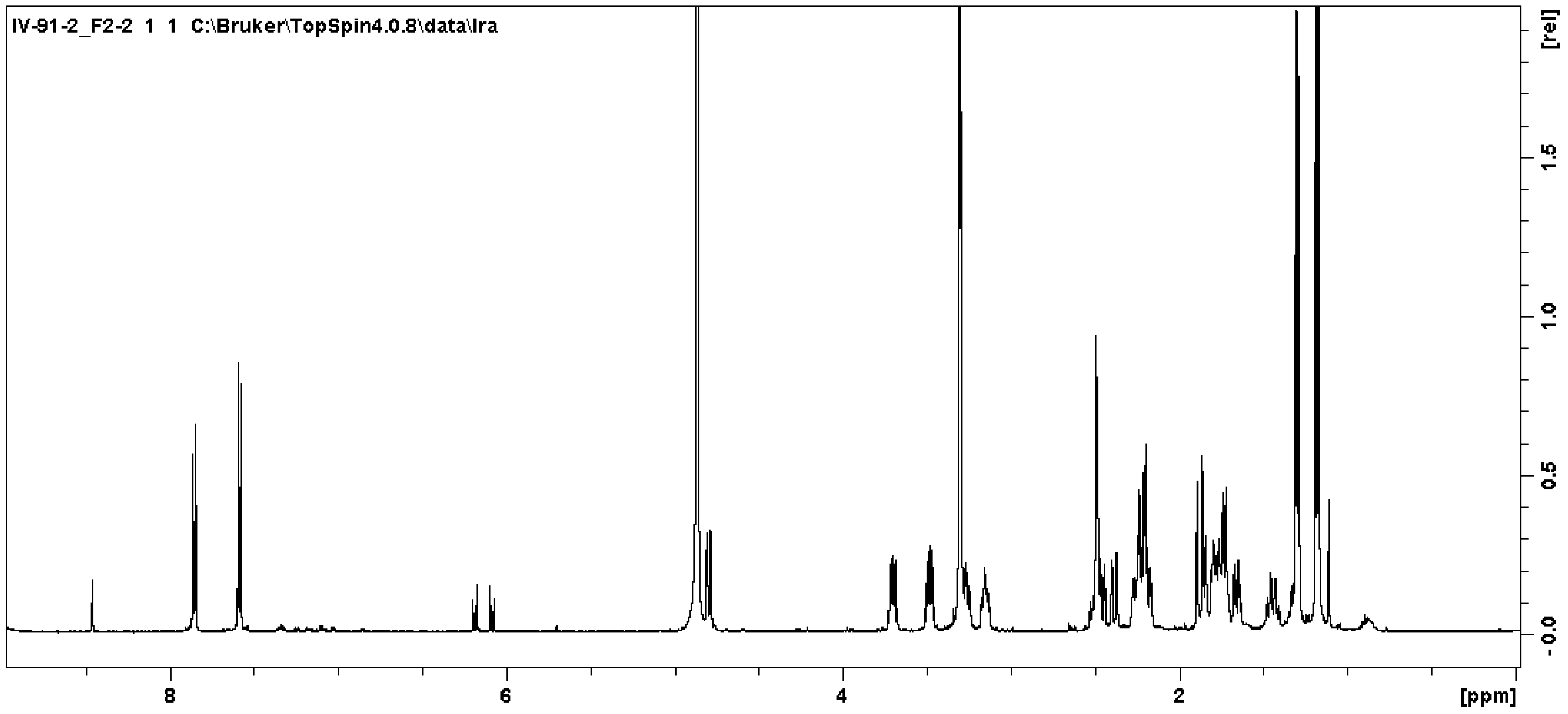

Figure S11. ^{1}H spectra of Baikalomycin B in CD_3OD.

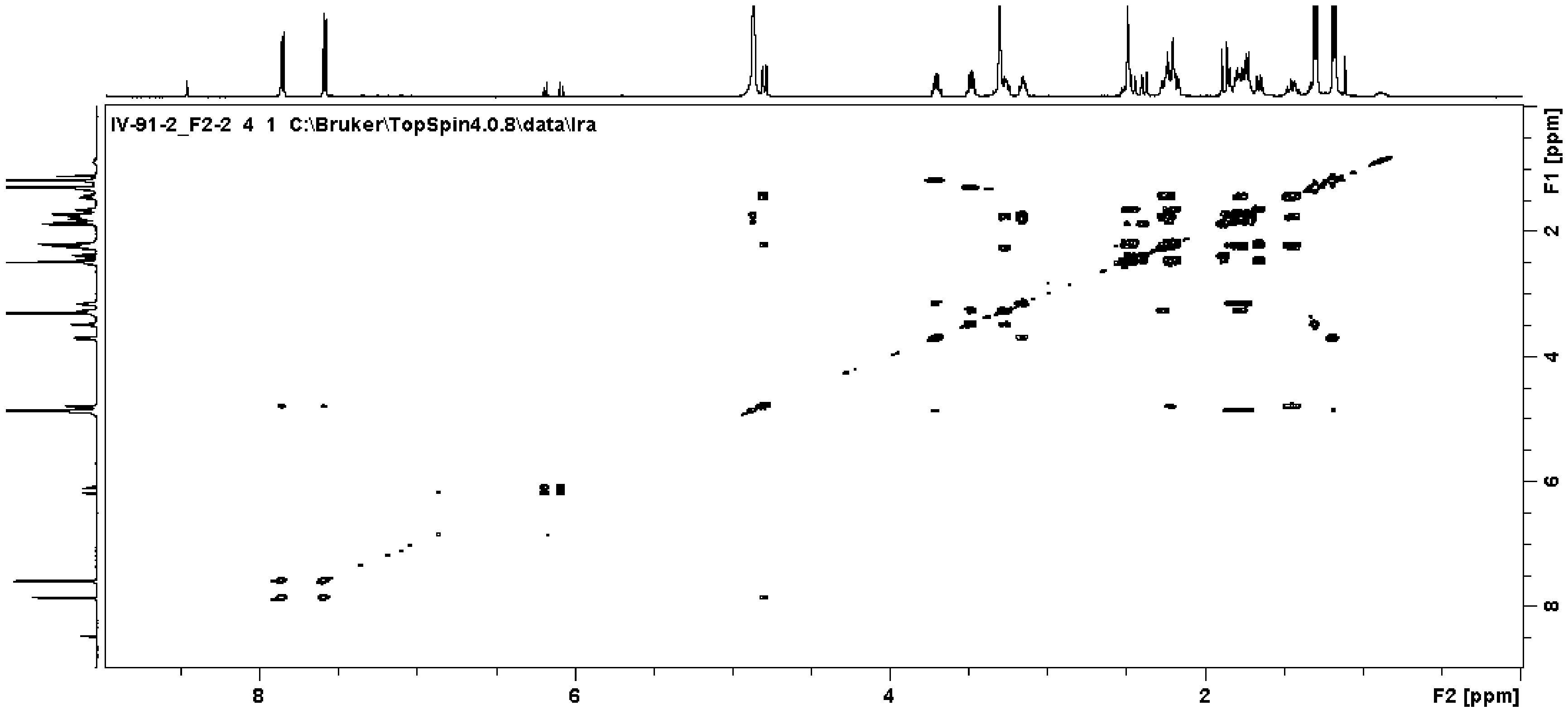

Figure S12. ^{1}H-^{1}H-Cosy spectra of Baikalomycin B in CD_3OD.

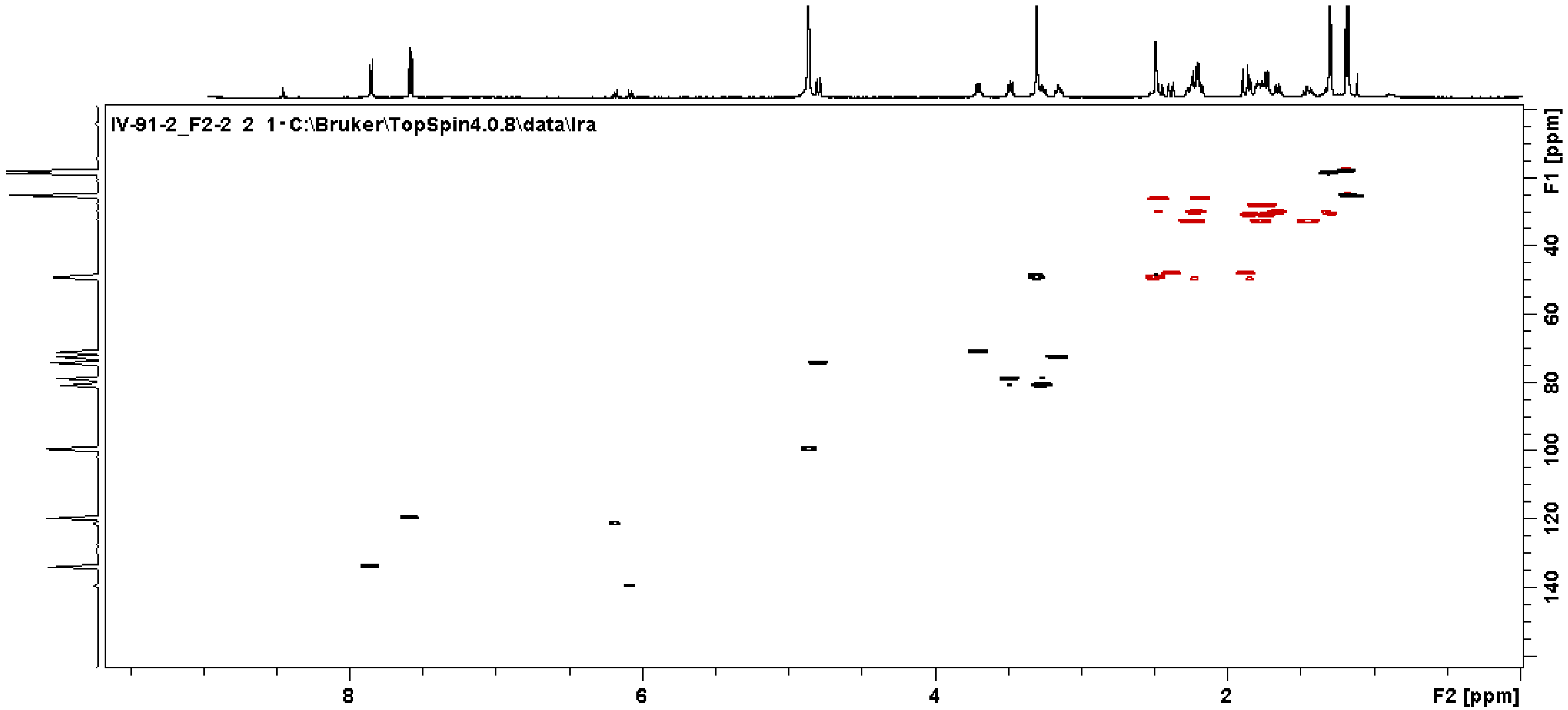

Figure S13. HSQC spectra of Baikalomycin B in CD_3OD.

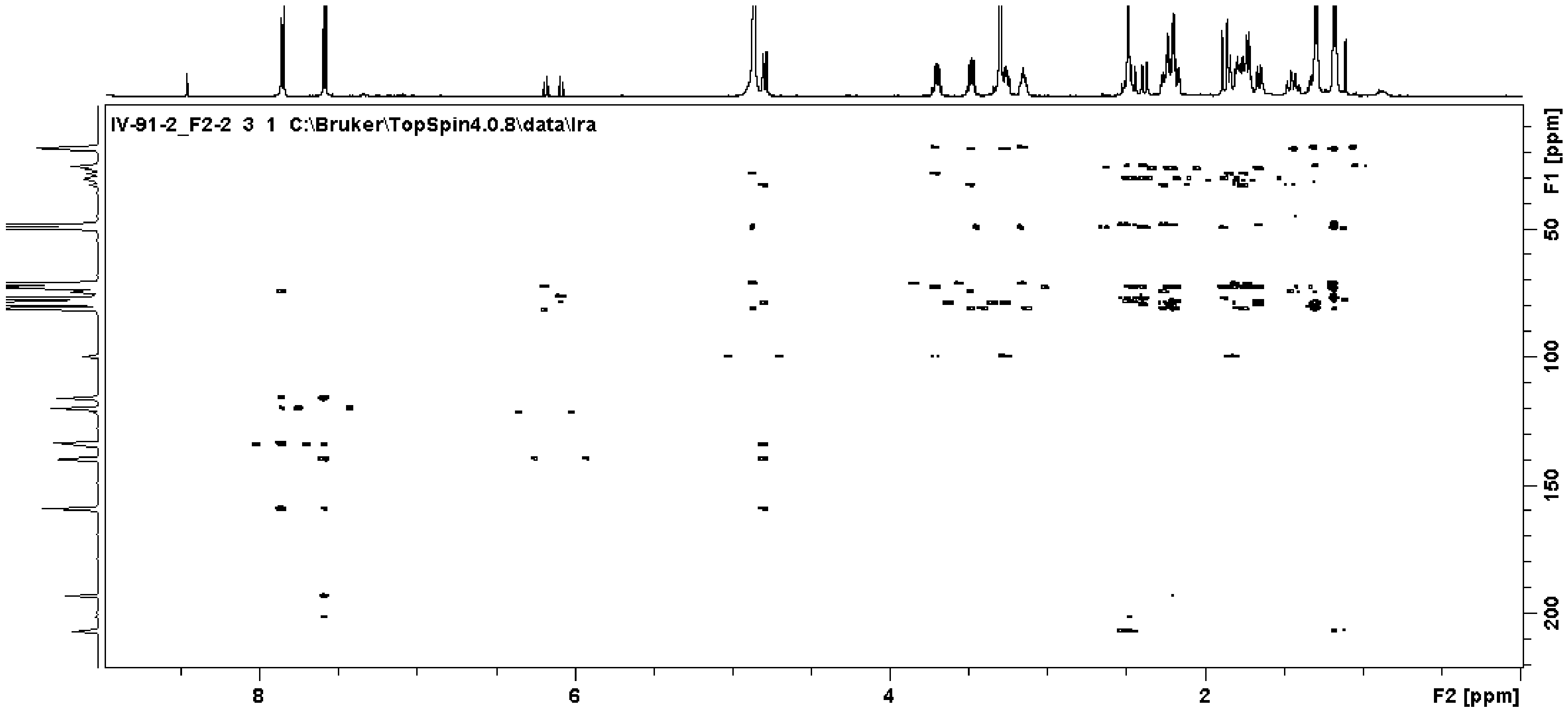

Figure S14. HMBC spectra of Baikalomycin B in CD_3OD.

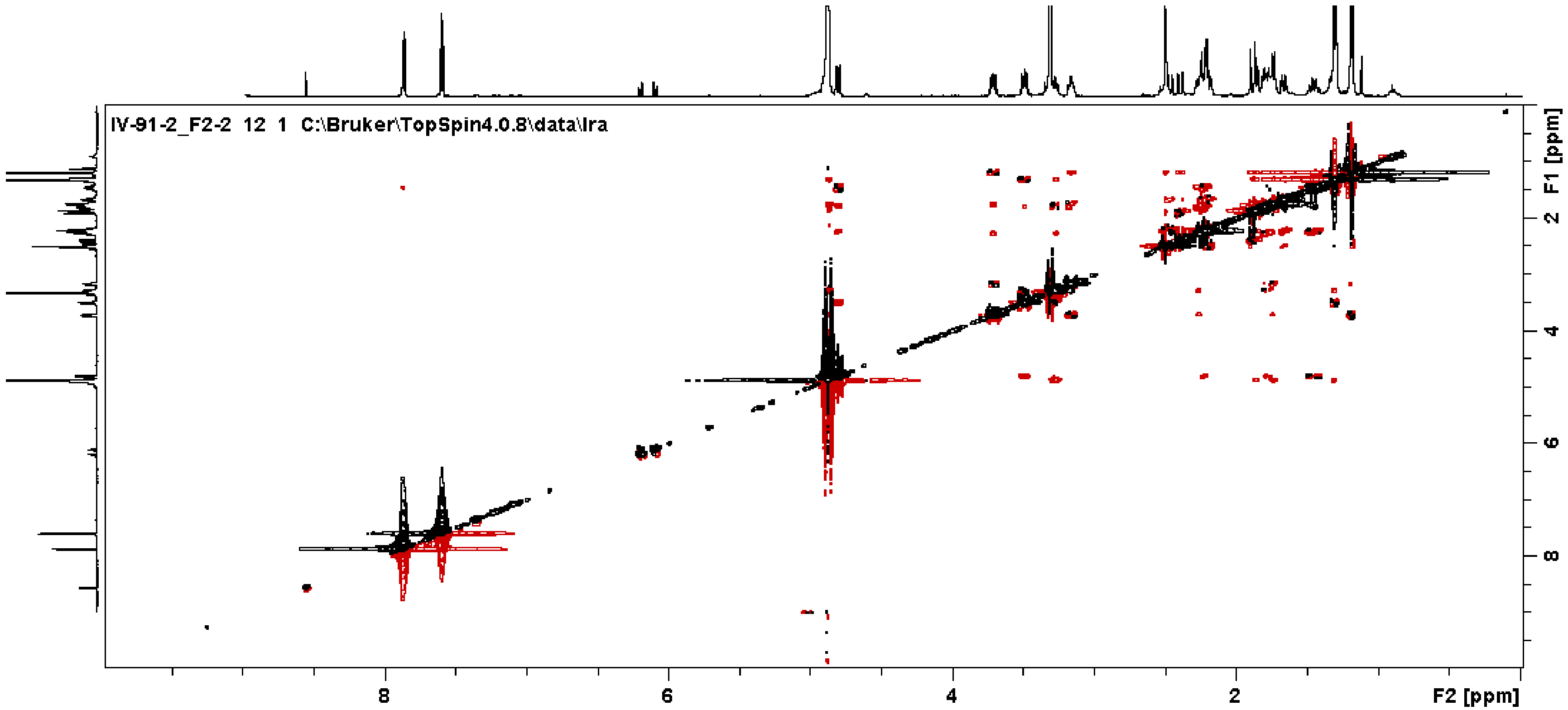

Figure S15. ROESY spectra of Baikalomycin B in CD_3OD.

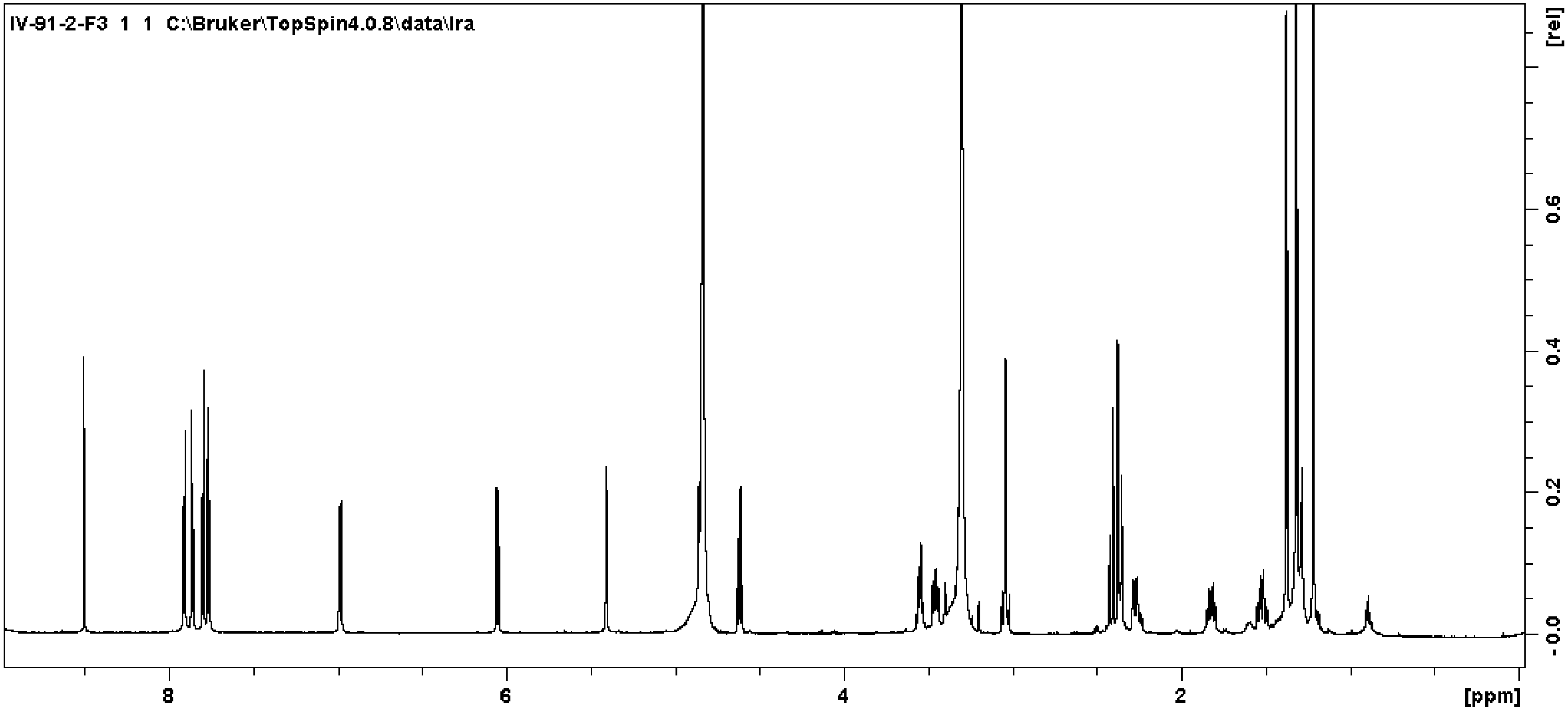

Figure S16. ^{1}H spectra of Baikalomycin C in CD_3OD.

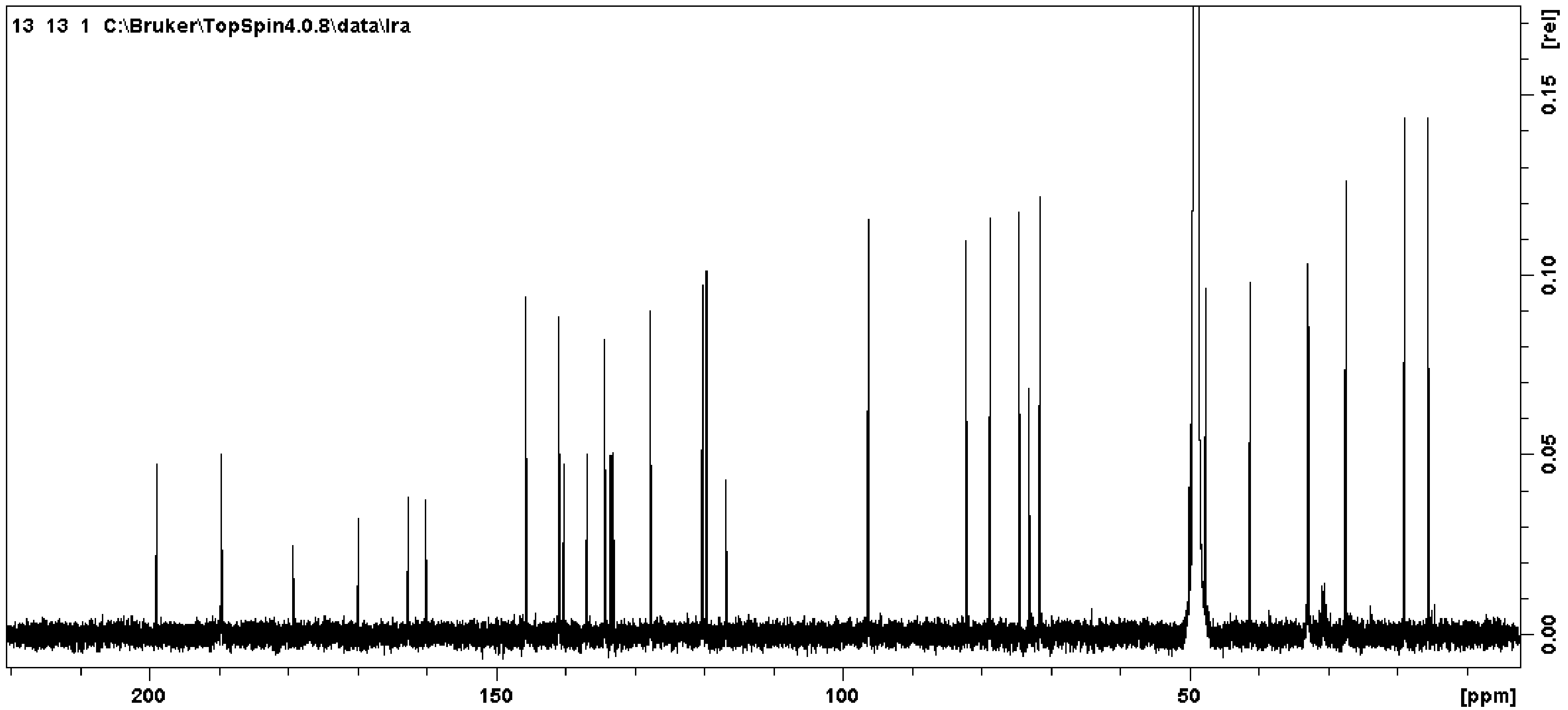

Figure S17. ^{13}C spectra of Baikalomycin C in CD_3OD.

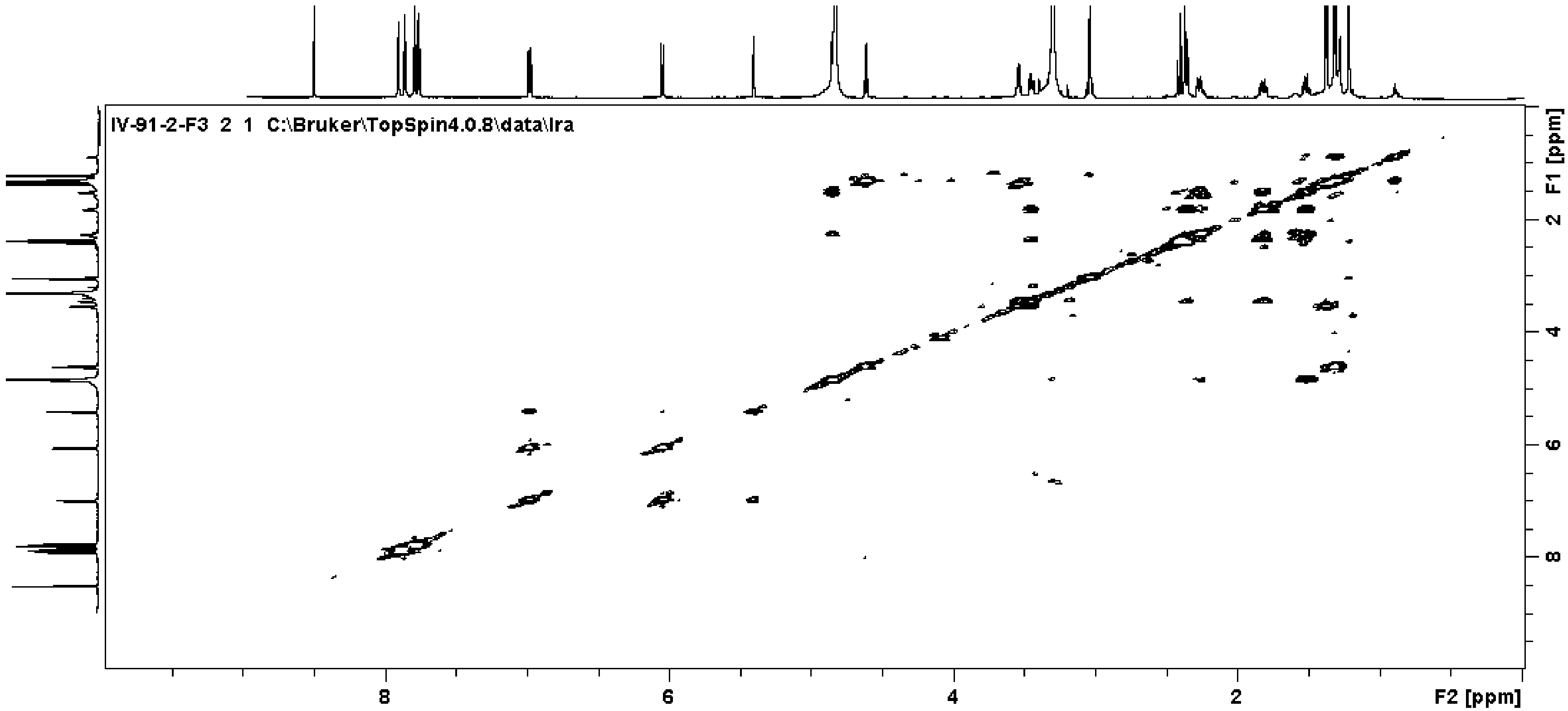

Figure S18. ^{1}H-^{1}H-Cosy spectra of Baikalomycin C in CD_3OD.

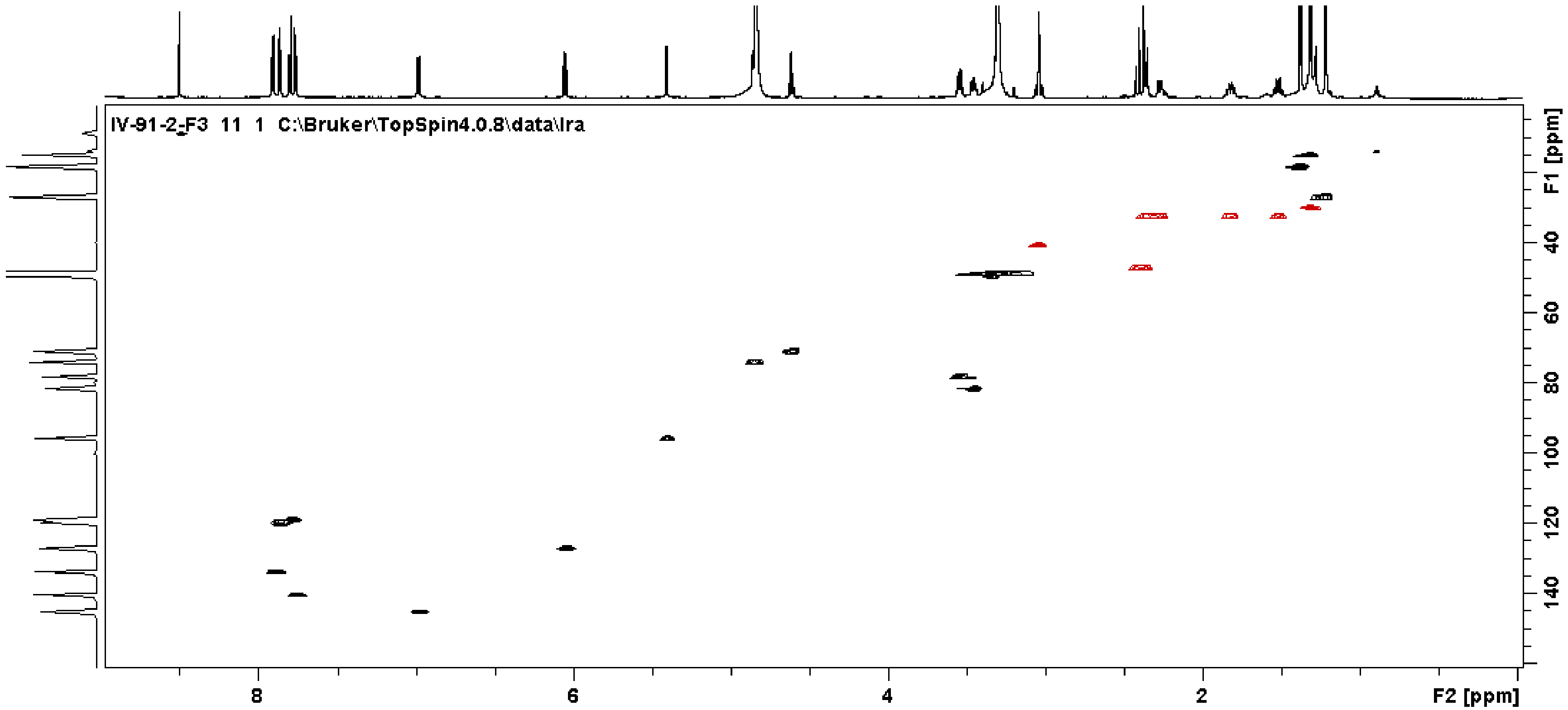

Figure S19. HSQC spectra of Baikalomycin C in CD_3OD.

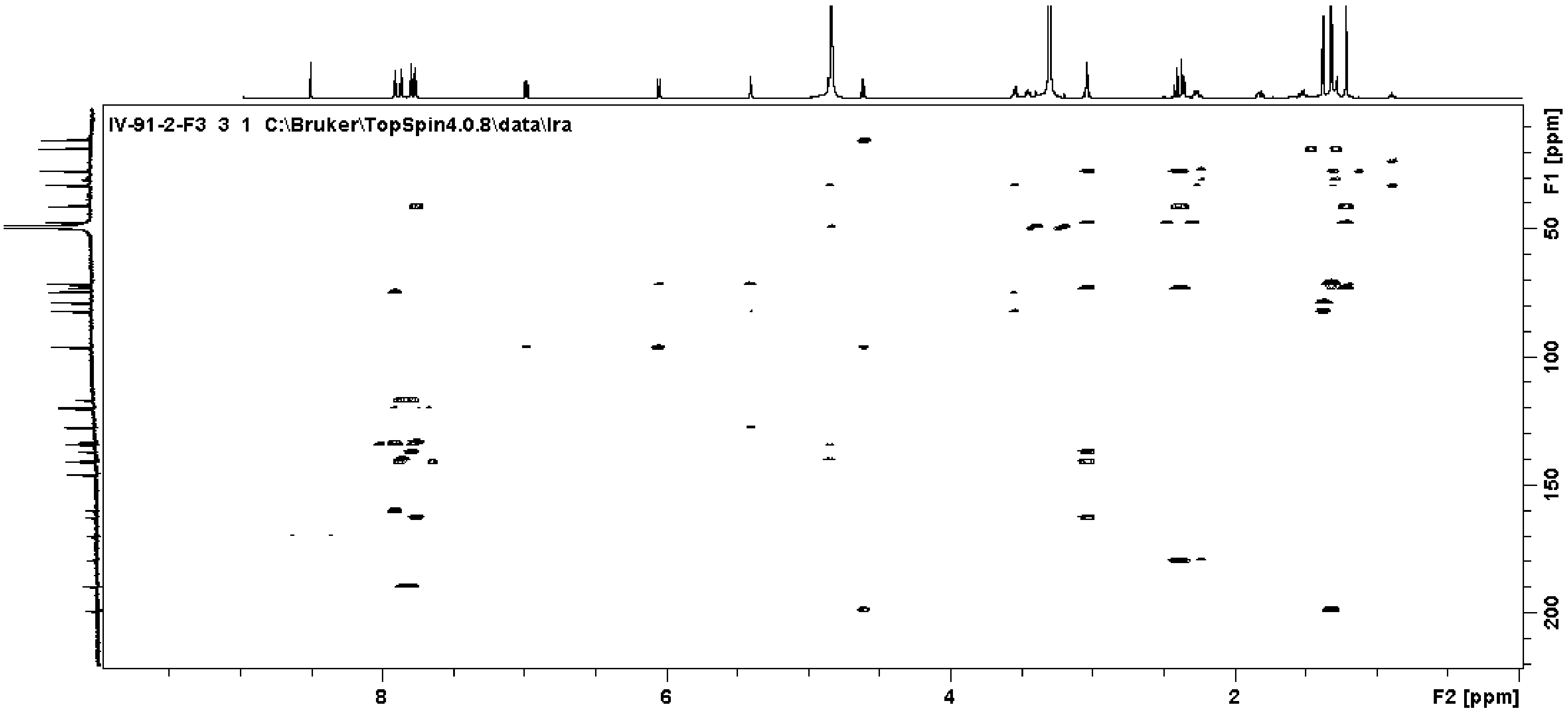

Figure S20. HMBC spectra of Baikalomycin C in CD_3OD.

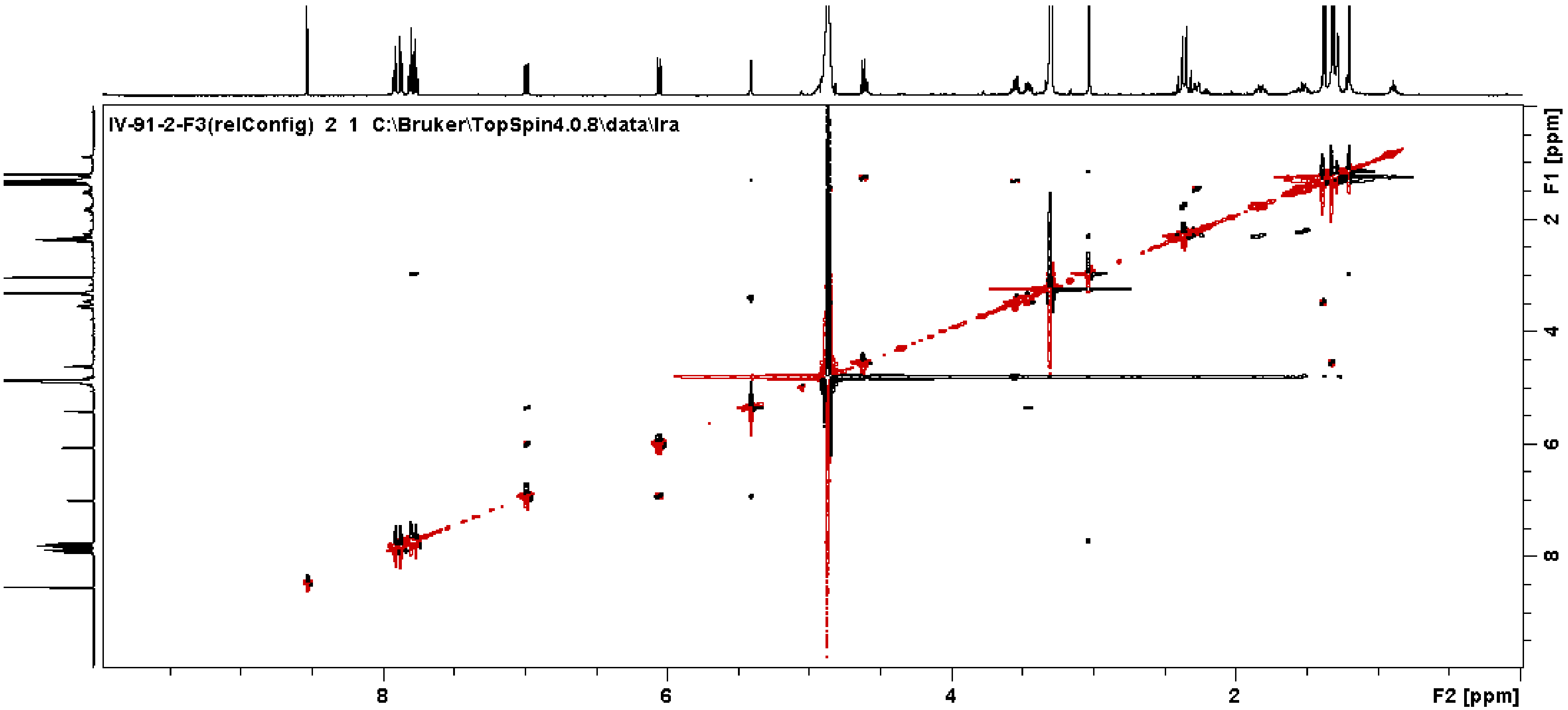

Figure S21. ROESY spectra of Baikalomycin C in CD_3OD.

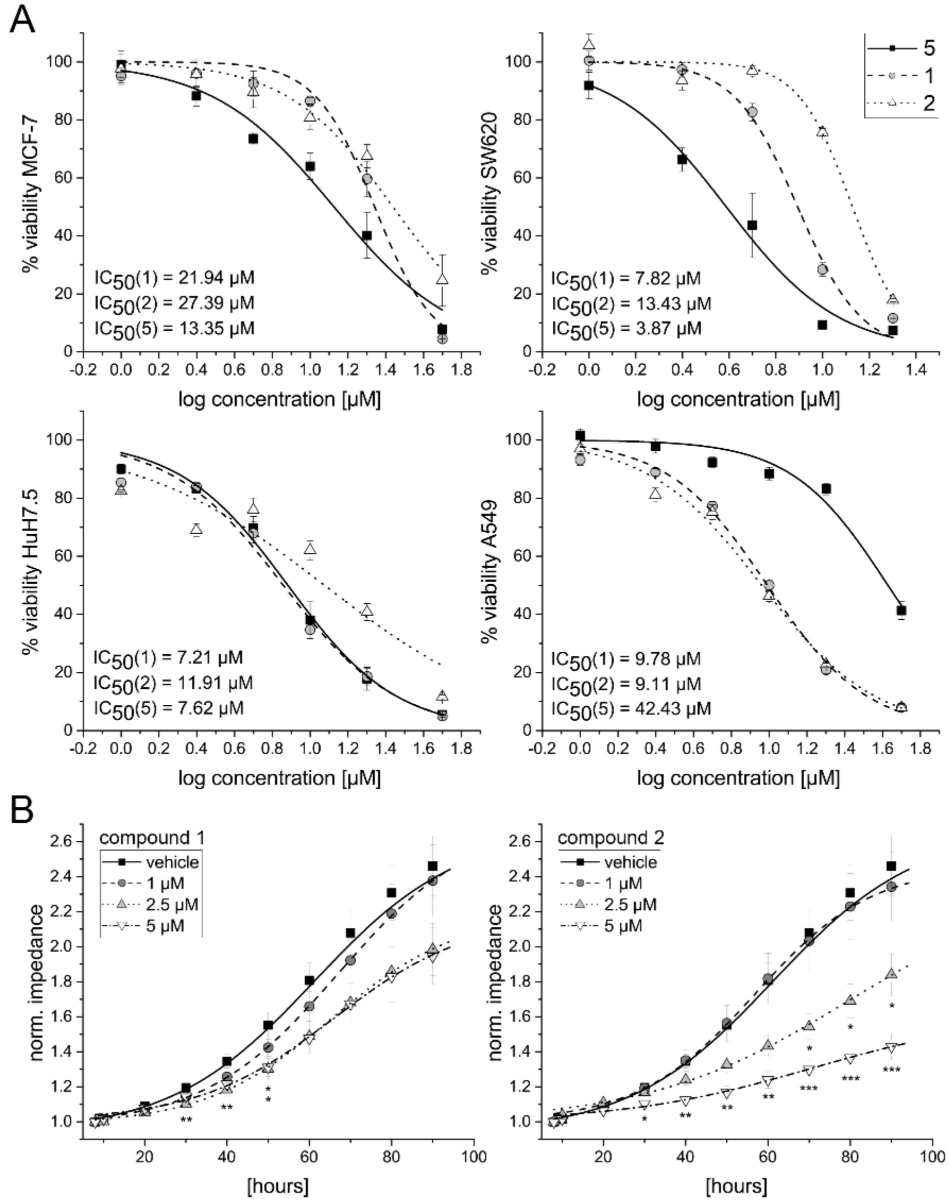

Figure S22. Anticancer activities. (A) Cancer cell viability was measured after 48 h treatment in an MTT assay normalized to the respective diluted solvent DMSO control (n=3, quadruplicates). (B) Cell proliferation was measured using the electric cell-substrate impedance sensing (ECIS®) system. Cells were treated 5 h after seeding. Control cells (vehicle) were treated with the diluted solvent DMSO. Impedance was normalized to values at 7 h after seeding (n=3, quadruplicates). Graphs show mean values ± SEM every ten hours and non-linear regression curves for all time points. * $p < 0.05$, ** $p < 0.01$ ***, $p < 0.001$ compared to the control at the respective time point.

Table S6. Distribution of secondary metabolite gene clusters within genome of *Streptomyces* sp. IB201691-2A based on antiSMASH analysis.

**Cluster type**	**Number of clusters**
NRPS	6
NRPS, phenazine	1
NRPS, nucleoside	1
PKS I	2
PKS I-NRPS	3
hglE-KS	1
PKS II	1
PKS III	3
Melanin	2
Siderophore	4
Terpene	9
Bacteriocin	2
Cyanobactin	1
Ectoine	1
Phenazine	1

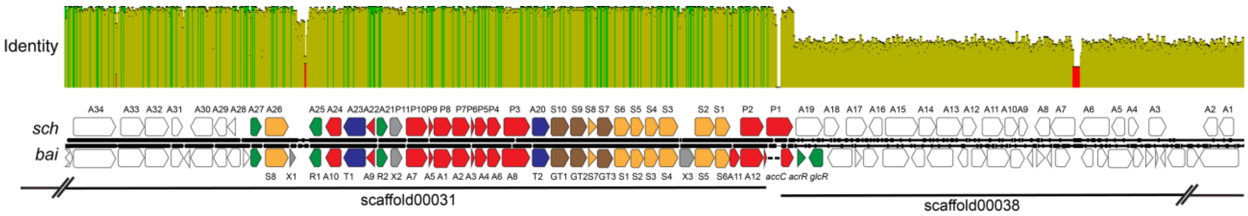

Figure S23. Pairwise alignment of *bai* biosynthetic gene cluster from *Streptomyces* sp. IB201691-2A with *sch* Sch47554/47555 biosynthesis gene cluster from *Streptomyces* sp. SCC-2136. Scaffolds of *Streptomyces* sp. IB201691-2A genome covering entire *bai* gene cluster are shown.

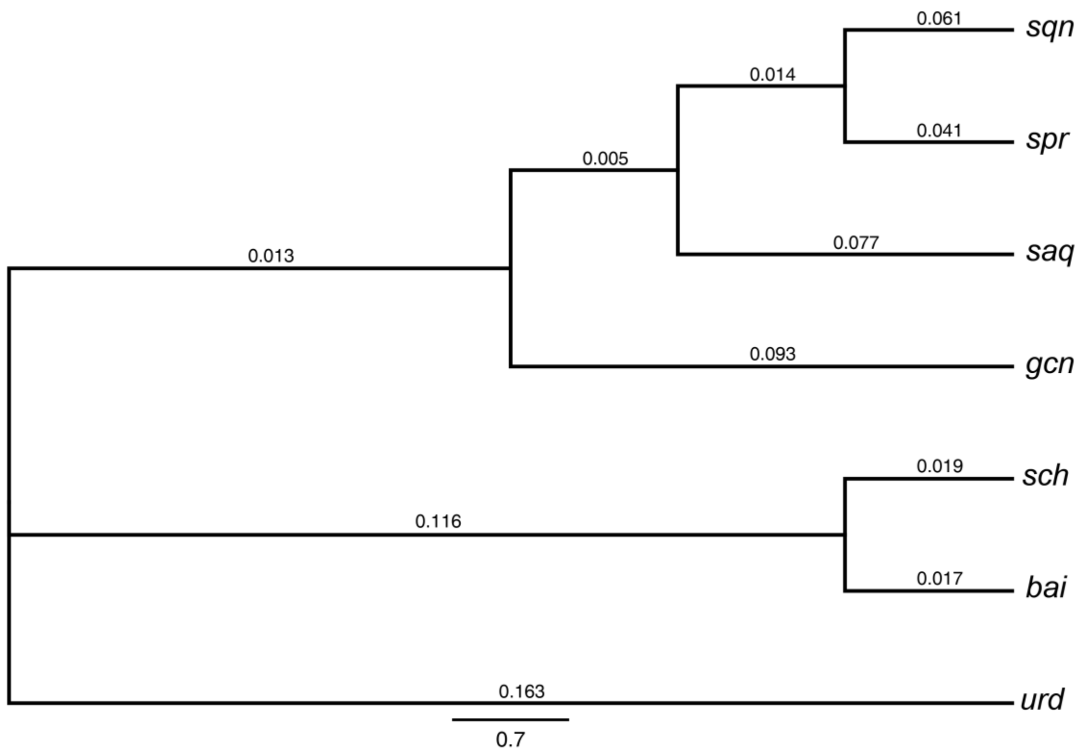

Figure S24. Evolutional relationship between biosynthetic clusters.

sqn - saquayamycin gene cluster from *Streptomyces* sp. KY40-1, *spr* - saprolomycin gene cluster from *Streptomyces* sp. TK08046, *saq* - saquayamycin gene cluster from *S. nodosus* ATCC4899, *gcn* - grincamycin gene cluster from *S. lusitanus* SCSIO LR32, *sch* - Sch47554/47555 gene cluster from *Streptomyces* sp. SCC-2136, *bai* – baikalomycin gene cluster from *Streptomyce*s sp. IB201691-2A, *urd* – urdamycin gene cluster from *Streptomyces fradiae* Tu2717.

Table S7. Baikalomycin gene cluster (*bai*) with predicted functions for each gene and homology to similar genes found within other *Streptomyces* sp.

ORF	Predicted function	Homologous	% Identity	Sequence ID
baiS8	flavin-dependent oxidoreductase	*SchA26, Streptomyces* sp. SCC 2136	97	CAH10126.1
baiX1	unknown	*Streptomyces* sp. TK08046	61	BAV17022.1
baiR1	repressor-response regulator	*lndI, Streptomyces globisporus*	66	AAU04840.1
baiA10	NADH:flavin oxidoreductase	*Streptomyces* sp. AM-2504	76	TBO55069.1
baiT1	transporter	*Streptomyces* sp. HG99	86	PIB12227.1
baiA9	flavin-reductase	*Streptomyces bingchenggensis* BCW-1	80	ADI11037.1
baiR2	TetR family transcriptional regulator	*Streptomyces* sp. NBS 14/10	75	OXL27454.1
baiX2	unknown	*SaqP, Micromonospora* sp. Tu 6368	48	ACP19350.1
baiA7	hydroxylase	*UrdE, Streptomyces* fradiae Tü2717	78	CAA60567.1
baiA5	cyclase/aromatase	*SchP4, Streptomyces* sp. SCC 2136	99	CAH10113.1
baiA1	ketoacyl synthase alpha	*SchP6, Streptomyces* sp. SCC 2136	99	CAH10117.1
baiA2	chain length factor	*SchP7, Streptomyces* sp. SCC 2137	98	CAH10116.1
baiA3	acyl- carrier protein	*SchP8, Streptomyces* sp. SCC 2138	97	CAH10115.1
baiA4	ketoreductase	*SchP5, Streptomyces* sp. SCC 2136	98	CAH10114.1
baiA6	cyclase/aromatase	*SchP9, Streptomyces* sp. SCC 2136	99	CAH10113.1
baiA8	oxygenase-reductase	*UrdM, Streptomyces fradiae* Tü 217	85	AFU51427.1
baiT2	transporter	*PgaJ, Streptomyces sp.* PGA64	65	AAK57531.1
baiGT1	O-glycosyltransferase	*SchS10, Streptomyces* sp. SCC 2136	97	CAH10110.1
baiGT2	O-glycosyltransferase	*SchS9, Streptomyces* sp. SCC 2136	98	CAH10109.1
baiS7	NDP-hexose 3,5-epimerase	*UrdZ1, Streptomyces fradiae* TU 2717	71	AAF00208.1
baiGT3	C-glycosyltransferase	*SchS7, Streptomyces* sp. SCC 2136	98	CAF31363.2
baiS1	glucose-1-phosphate thymidylyltransferase	*Streptomyces* sp. MJM1172	77	OKI61982.1
baiS2	NDP-glucose 4,6-dehydratase	*Streptomyces* sp. SCC 2136	98	CAF31365.1
baiS3	dTDP-6-deoxy-L-talose-4-dehydrogenase	*Streptomyces hundungensis*	50	AYG82949.1
baiS4	dTDP-4-amino-4,6-dideoxy-D-glucose transaminase	*Streptomyces bingchenggensis* BCW-1	83	ADI09991.1
baiX3	unknown	*Streptomyces* sp. TK08046	76	BAV17015.1
baiS5	NDP-hexose 2,3-dehydratase	*SaqS, Micromonospora* sp. Tu 6368	73	ACP19377.1
baiS6	glucose-fructose oxidoreductase	*Streptomyces nodosus*	78	AJE43663.1
baiA11	phosphopantheine-transferase PgaX	*Streptomyces dengpaensis*	77	AVH55999.1
baiA12	methylmalonyl-CoA carboxyltransferase	*Streptomyces scabiei*	90	KFF99397.1

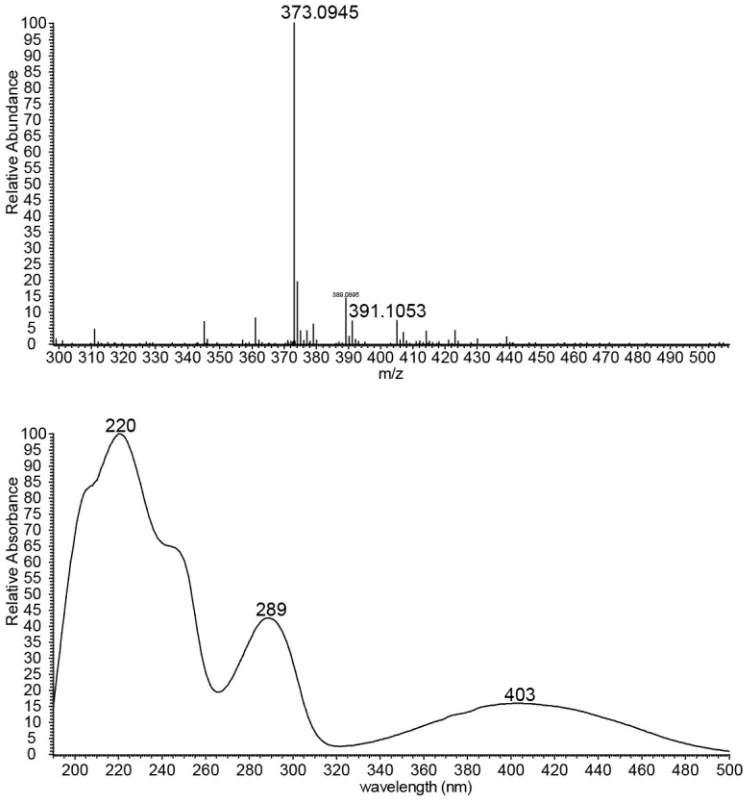

Figure S25. Mass and UV absorption spectra of aglycone compound X1 found in the extract of the mutant strain *Streptomyces* sp. IB201691-2AΔGT3 and IB201691-2AΔGT2.

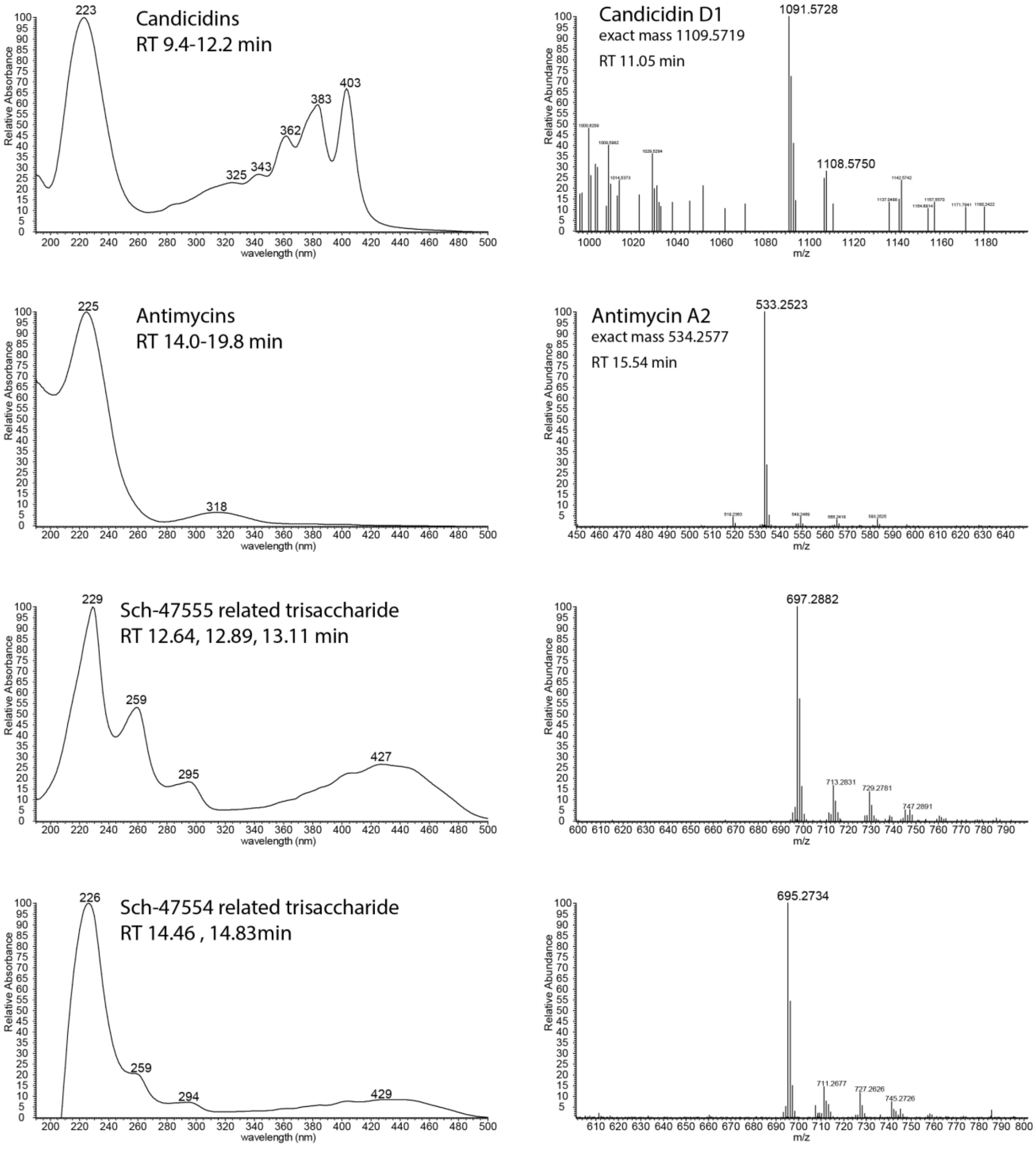

Figure S26. Baikalomycin trisaccharides found in the extract of cultivated host strains *S. albus* J1074/p8-13bai which contains the baikalomycin gene cluster closely related to SCH-47554/47555 gene cluster. Candicins and antimycin compounds were accumulated in *S. albus* J1074.

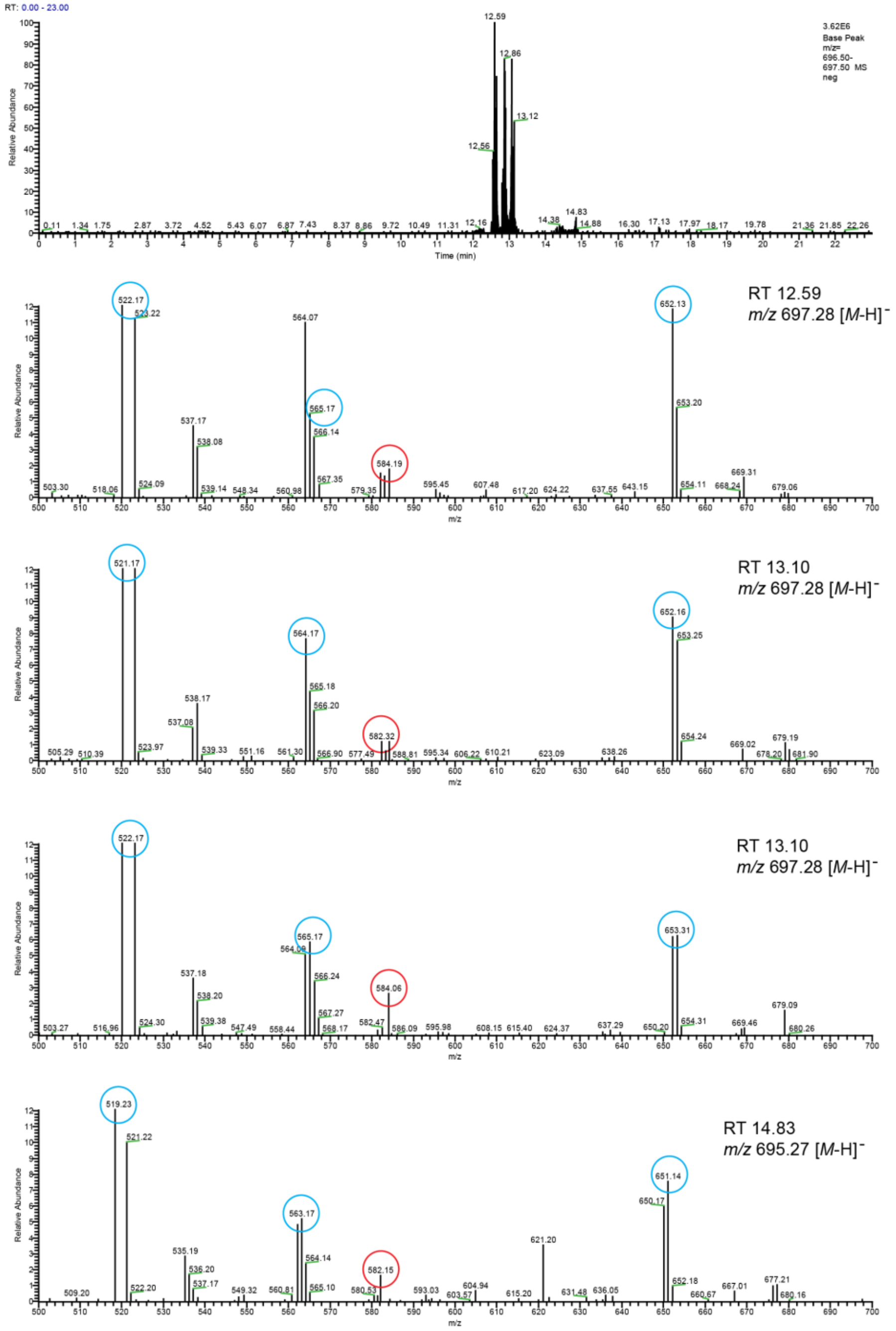

Figure S27. MS/MS fragmentation of Baikalomycins trisaccharides found in the extract of cultivated host strains *S. albus* J1074/p8-13bai which contains the baikalomycin gene cluster closely related to SCH-47554/47555 gene cluster. Fragmentation of compound with m/z 697.2882 [M-H]$^-$ (RT 12.64, 12.89 and 13.11 min) (A) and of compound with m/z 695.2734 [M-H]$^-$ (RT 14.83 min) (B). Loss of 113 Da coincides with the sugar cinerulose, loss of 115 da coincides to the sugar amicetose or rhodinose.
